# “The Harder One Tries …”: Findings and Insights From the Application of Covert Response Pressure Assessment Technology in Three Studies of Visual Perception

**DOI:** 10.1177/2041669520913319

**Published:** 2020-04-20

**Authors:** Myron Tsikandilakis, Persefoni Bali, Giannis Haralabopoulos, Jan Derrfuss, Peter Chapman

**Affiliations:** School of Psychology, University of Nottingham; School of Medicine, Faculty of Medicine and Health Sciences, University of Nottingham; School of Psychology, University of Nottingham; School of Computer Science, University of Nottingham; School of Psychology, University of Nottingham

**Keywords:** confidence, difficulty, physiology, assessment, implicit

## Abstract

In this article, we present a force measuring method for assessing participant responses in studies of visual perception. We present a device disguised as a mouse pad and designed to measure mouse-click-pressure and click-press-to-release-time responses by unaware, as regards to the physiological assessment, participants. The aim of the current technology, in the current studies, was to provide a physiological assessment of confidence and task difficulty. We tested the device in three experiments. The studies comprised of a gender-recognition study using morphed male and female faces, a visual suppression study using backwards masking, and a target-search study that included deciding whether a letter was repeated in a subsequently presented letter string. Across all studies, higher task difficulty was associated with higher click-release-time responses. Higher task difficulty was, intriguingly, also associated with lower click pressure. Higher confidence ratings were consistently associated with higher click pressure and shorter click-release time across all experiments. These findings suggest that the current technology can be used to assess responses relating to task difficulty and participant confidence in studies of visual perception. We suggest that the assessment of release times can also be implemented using standard equipment, and we provide manual and easy-to-use code for the implementation.

## Introduction

Previous research has suggested that explicit assessment tasks are subject to limitations. For example, participants in contemporary psychological studies come frequently from science-aware student, and academic and health-related professional groups. Their responses to explicit assessments can be subject to self-presentation strategies. These strategies have personal and social dimensions. These include the presentation of the self in a positive (Bareket-Bojmel et al., 2016), or expected to be perceived as positive, way (Dietrich et al., 2017). These also include professional fitness-to-practise self-presentation strategies, such as presenting oneself in a way that will not be perceived as inconveniencing professional employability (Kopera et al., 2015).

In addition to these, previous research has suggested that participants could themselves be unaware or in conflict as regards to reporting their experiences (Greenwald et al., 1998). According to this argument, participants could be biased in explicitly reporting their experiences due to introspective limits, such as antagonistic dual processing of knowledge (e.g., perception concerning a negative characteristic) and personal motivations (e.g., the intent to behave in a sociably acceptable manner; for a comprehensive review in this subject, see Strack & Deutsch, 2004). Previous research has also suggested that some information could be subject to preconscious or unconscious processing and, therefore, that the participants could be unable to report or less accurate in reporting their experience of these information when enquired using an explicit task (Dehaene et al., 2006; but see also Tsikandilakis et al., 2018, 2019c).

To address these possibilities, several contemporary psychologists have proposed alternative methodologies that are, arguably, suitable for the implicit assessment of cognition, behaviour, and emotion, such as responses that a participant will not be able or willing to report when asked using an explicit assessment. For example, Schnabel et al. (2008) proposed the use of the implicit association test (see Greenwald et al., 1998) and suggested that the time to respond to a task with a keyboard press that is conditioned to refer to oneself (e.g., me) is longer when an incompatible to a self-presentation strategy word (e.g., unsociable) is subsequently presented and assigned the same keyboard component.

Further experimental assessments include explicit physiological measurements of purportedly automatic and involuntary physiological responses to emotional stimuli, such as subcutaneous sweating and heart-rate changes (van der Ploeg et al., 2017). The aim of measuring these responses is to directly record what a participant is experiencing during a task or trial on an emotional-physiological level (Bradley & Lang, 2000). Experimental approaches in this area also include implicit or explicit camera monitoring that aims to assess and interpret the facial responses of the participants using automated facial-emotional recognition software (Lewinski, 2015). The aim of these applications is to measure posttrial and engagement task responses directly and without relying on self-reports.

Previous research has suggested that not only emotional responses could be assessed implicitly and in the area of engagement task response assessment, but previous research has also suggested that participants also have self-presentation strategies (Strack & Deutsch, 2004). These include rating the task difficulty of an engagement task high and downrating the confidence for their responses due to conservative self-presentation strategies, such as presenting oneself as attempting to perform at the best of their ability. Previous research has also suggested that certain participants downrate task difficulty and overstate their response confidence to come across as overachievers (Hellmann, 2016; Kosakowska-Berezecka et al., 2017).

In the current studies, we adjust previous technologies relating to force-grip pressure in pharmaceutical (Perkins et al., 2009) and rehabilitation studies (Allgöwer et al., 2017) and adjust previous work on finger-sensor force-assessment technology from human (Akamatsu & MacKenzie 1996; Akamatsu & Sato, 1994; Kaklauskas et al., 2009) and animal studies (Deacon, 2013). In these studies, researchers have reported that high task difficulty resulted in greater hesitation and, therefore, lower force pressure due to the experience of uncertainty as regards the correct answer. Previous research has also reported that, for the same reasons, higher confidence in a response resulted in higher force pressure and lower response times. This line of research found very high effect sizes for physiological responses to task difficulty (see, e.g., Bilalpur et al., 2017; Campanella et al., 2001).

We use a seemingly innocuous device that is part of an individual’s everyday professional and domestic life activities (Schunk & Greene, 2017). We present a force pressure measuring device disguised as a mouse pad. The device was designed to assess physiological responses in tasks that varied in difficulty (Hellmann, 2016; Kosakowska-Berezecka et al., 2017). The device was also applied to assess the association between self-reports for response confidence and click-release time and force pressure (Hellmann, 2016; Kosakowska-Berezecka et al., 2017). We applied strict criteria for assessing click-release time and force pressure, such as the implicit assessment of participant responses, the application of individualized *z* scores for force pressure assessment, the strict exclusion of outliers for high and low force pressure responses, and the exclusion of possibly unintended click-responses that were associated with a high threshold of movement-related artifacts, such as bilateral mouse movements at or higher than 60 Hz (16.67 ms; see also Methods section: Force-Pad Specifications).

With these methods, we assess mouse-click force-pressure responses and click-release time in engagement tasks that required a mouse response. The initial application of this technology was designed to implicitly assess task difficulty and response confidence in masking experiments and explore possible evidence for subliminal processing (Brooks et al., 2012). This initial aim was in line with suggestions for further research in previous publications relating to physiological responses to masked emotional faces (see, e.g., Tsikandilakis et al., 2018). In the current article, we were able to expand the testing of the device. We tested, using the device, for task difficulty and response confidence in gender morphing perception tasks because previous research has suggested that gender perception is subject to explicit assessment biases relating to cultural and social norms (Burr, 2015) and that responses to nonbinary gender categorization could be explored using an implicit assessment of physiological responses to gender characteristics (Hyde et al., 2019). We also test the device in a letter-search experimental task because previous research relating to linguistic processing (Ellis, 2002; Jurafsky, 2000) has suggested that implicit (Yablonski et al., 2017) and physiological responses (Suslow et al., 2015) could be more accurate indicators of task difficulty compared with self-reports (Baumeister et al., 2007). These two additional experiments were added to the current stages to explore whether the current technology could be applied in these areas (Kim et al., 2017; Ryoo et al., 2018).

Our aim in these studies was to explore whether force-plate technology could be used to assess task difficulty and response confidence. Our hypotheses across all experimental stages were that higher task difficulty during the engagement tasks will cause higher uncertainty and hesitation as regards the correct response and be associated with longer click-release times and lower force-pressure responses. We also hypothesized that, for the same reason, high levels of response confidence will be associated with short click-release times and high force-pressure responses.

## Methods: Force-Pad Specifications

### 

**Figure 1.**
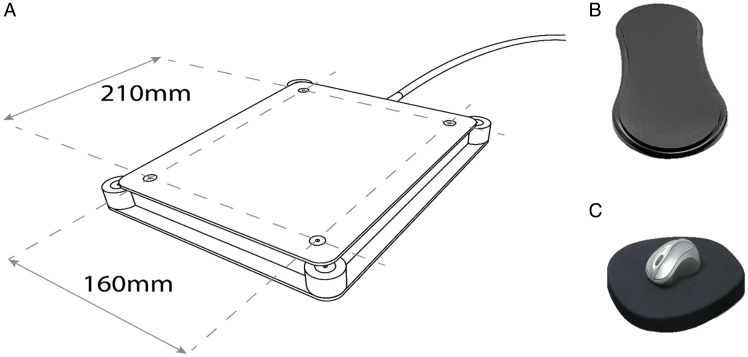
Graphic Illustrations of the Force-Pad Device. (A) The participant’s right hand was rested upon a 45- to 20- to 3-cm forearm support. (B) The device was adjusted to a solid wooden surface, and the wiring was covert via a desk apparatus beneath a custom-made ergonomic nonslip rubber-based mouse mat. The mat covered the working surface and the machine edges of the device. An ergonomic nonslip wireless Logitech M590 mouse was used for participant responses. (C) The prototype for the device was developed by the first author. The experimental version was manufactured by PSYAL.

The device was created to look like a mouse pad. It was disguised with a custom-designed nonslip rubber-based mouse mat that covered the entire surface and the edges of the machine (see Figure 1). The force pad included a working measurement area of 210 to 160 mm. It was mechanically coupled to four 5 kgf load cells. The signals from these cells were amplified and converted to a digital signal via a 24-bit analog-to-digital converter. The device operated at 120 Hz with a minimum force accuracy of 0.01 grams (0.00098 Newtons) and a maximum filtered pressure force of 5 kgf. The device included its own parallel-port input and interfaced with the stimulus-presenting computer using a USB 2.0. During the testing, the participant’s right hand (see Supplementary Material 2.1) was rested upon a 45- to 20- to 3-cm forearm support, and the participants operated the mouse using the forearm support to control for movement artefacts. Participants’ responses were computed via drift filtering and mapping the location of the mouse to an on-screen visual matrix. Subsequently, a load-cell filtering protocol calculated and subtracted the mouse weight, the mouse mat weight, and the hand weight of the participant applied after each bilateral movement from the force applied every time the participant responded using a mouse click (see Supplementary Material 7.1). To control for outliers due to individual strength, each participant was asked during a training session to press 20 times on one of two paralleled positioned and interchangeably designated, with the words *Press Here*, on-screen boxes (pt. 0.73 (H) to 2.33 (W); interval space: pt. 1.62 (W)). The *z* scores for click-pressure responses per participant were calculated. The force applied during the main experimental stages was measured in units of standard deviations from the performance in the training phase (see Supplementary Material 1.1 to 1.3). Conversely, click-release time was measured in milliseconds according to the available refresh rate of the force-pad device (120 Hz; 8.33 ms). It was then converted to rounded two decimal intervals of a second as the duration between an unambiguous-filtered (≥85.03 grams; ≤781.62 grams)^1^ click pressure and an equal unambiguous-filtered release of the applied force. The presence of a response was additionally confirmed using a self-developed parallel-port output script (see Supplementary Material 7.1). Responses that were higher or lower than two standard deviations of the mean for pressure strength for individual performance and responses that indicated drift frequency that could be related to movement artefacts, that is, bilateral drift frequency ≥ 60 Hz (one bilateral drift per 16.67 ms) for overall drift duration *x* ≥125 ms, were excluded from the analysis (see Supplementary Material 7.1). Using this protocol, click pressure and click-release time were recorded in each study. Click-pressure and click-release-time responses were measured and analysed exclusively and only for the first binary, gender (male/female), or face-detection (yes/no) or letter-search (yes/no) task in each of the included studies. Their association to self-reports for participant confidence and methodological manipulations of task difficulty was explored.

**Figure 2.**
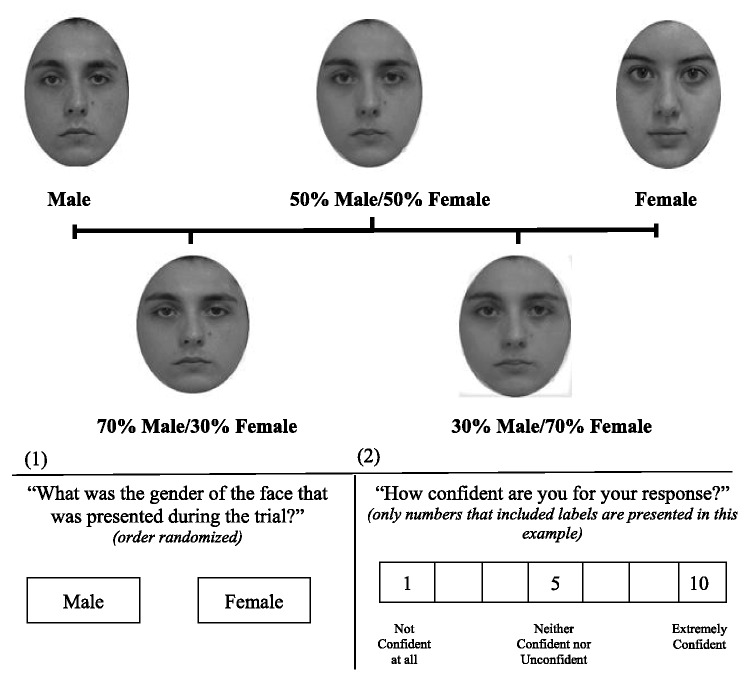
Example of Facial Merging and Engagements Tasks. Participants watched a face for 1 s with set (male, male–female, female) and varying gender characteristics (10% or 30% or 50% or 70% or 90%). They were afterwards assigned the engagement tasks illustrated in the figure: (1) and (2). Each engagement task was presented separately and in the described order. After each trial, a 7-s blank-screen interval was presented.

## Study 1: Gender Recognition

### Aims

The aim of the current study was to explore force-pad click-pressure and click-release-time responses to gender-ambiguous stimuli. We wanted to explore whether the current technology can be used to assess response confidence and task difficulty in morphing studies. The hypothesis during this stage was that faces with more ambiguous gender characteristics would result in higher task difficulty and, therefore, that participants could respond with longer click-release times and lower click pressure to these stimuli. We also hypothesized that ratings of confidence would be positively associated with click pressure and negatively associated with release time.

### Participants

A power calculation based on medium effect sizes (partial eta-squared = .06, *f* = .25) was performed. The result revealed that 36 participants would be required for *P*_(1–β)_ ≥ .95 (Faul et al., 2009). A total of 59 volunteers (28 female) participated in the current study. The exclusion criteria were current or previous DSM Axis I or II diagnosis and current or previous alcohol/drug abuse through self-reports. The participants were right-handed (see Supplementary Material 2.1). All participants had normal or corrected-to-normal vision. The participants were screened with the Somatic and Psychological Health Report Questionnaire (Berryman et al., 2012), and participants with scores above 1.0 were excluded from the analysis. Participants were screened with an online Alexithymia (2019) questionnaire, and participants with scores more than 94 that indicated possible alexithymia traits were excluded from the analysis. Participants were also screened with a hyperactivity-impulsivity questionnaire (Kooij & Francken, 2010), and participants whose scores indicated a possible hyperactivity-impulsivity disorder were excluded from the analysis; data from two participants were excluded. Data from three participants were excluded from the analysis due to movement-related artefacts (see Supplementary Material 7.1). The final population sample consisted of 54 (28 female) participants with mean age 29.54 (*SD* = 6.41). All participants gave consent for recordings before experiment and were fully debriefed concerning the precise nature of the physiological assessment after the experiment. The experiment was approved by the Ethics Committee of the School of Psychology of the University of Nottingham.

### Procedure

The facial stimuli used were taken from the dataset created by Gur et al. (2002). The stimuli included different male and female actors showing neutral expressions (*n* = 153). All the stimuli were adjusted for interpupillary distance, transformed to grey scale and resized to a standard 811 × 1,080 pixels resolution for software compatibility. Their luminescence was averaged using the SHINE MATLAB toolbox, and finally, they were spatially aligned and framed into pure white within a cropped circle (height: 6 cm, width: 4 cm).

All the stimuli were first assessed using Noldus 7.1. The analyses employed the Viola-Jones cascaded algorithm and an active appearance model with a 500-point Euclidean transformation to further eliminate static identification variability for image quality, lighting, background variation, and orientation. The software estimated the age range group of each actor in 5-year approximation intervals (e.g., 20–25, 25–30, 30–35, 35–40), the ethnic background of each actor, their gender, and their emotional expression. Faces that were misclassified in this assessment in comparison with their dataset labels were excluded (*n* = 4). To rigorously control for merging artefacts, all merged faces were matched for age interval and ethnic background, and they were validated for being of opposite gender. Actors with pronounced gender-specific appearance traits, such as facial hair, pronounced makeup, eyeliner, lipstick, and facial piercings, were excluded from the merging conditions.

### Experiments

Participants took part in a 5-min training stage. By the end of the training phase, they were asked whether they were ready to participate in the experiment. All participants replied positively. The experimental study consisted of two stages with order randomized. Participants were allowed a 5-min break between each stage. During the engagement tasks in this study, all text was presented in black on a white background using a clearly visible and standard font (Times New Roman), with a clearly visible font size (pt. 28). Each response-related part of text was boxed within a clearly visible and distinct selectable area with set dimensions (binary task: pt. 0.73 (H) to 2.33 (W); interval space: pt. 1.62 (W)). Participants were briefed during the training stage that they had the choice to restart the engagement task by pressing *space* in case of accidental miss-clicks; no instances of miss-clicks were reported.

In one stage, a fixation cross was presented for 2 (±1) s. After the fixation cross, a random male or female face or a merged male–female face with balanced male to female characteristics (50% merging layer) was presented for 1 s at fixation. A black and white pattern mask was then presented for 1 s at fixation. After the pattern mask, a blank screen was presented for 1 s. After the blank-screen interval, the participants were asked to decide the gender of the presented face (male/female) using the mouse. After this task, they were asked to rate how confident they were in their reply from 1 (*not confident at all*) to 10 (*extremely confident*) using the mouse. After each trial, a 7-s blank-screen interval was presented. A total of 20 male, female, and merged faces from different actors were presented during this stage.

In the other stage, the participants were presented with a fixation cross for 2 (±1) s. After the fixation cross, a random merged male–female face with varying male to female characteristics (10%, 30%, 50%, 70%, 90%) was presented at fixation. A black and white pattern mask was then presented for 1 s at fixation. After the pattern mask, a blank screen was presented for 1 s. After the blank-screen interval, the participants were asked from an on-screen message to decide the gender of the presented face (male/female) using the mouse. After this task, they were asked to rate how confident they were for their response from 1 (*not confident at all*) to 10 (*extremely confident*) using the mouse. After each trial, a 7-s blank-screen interval was presented. A total of five merged faces from different actors were presented for each interval (see Figure 2). No actor identity was repeated during the study. All the faces were merged, adjusted and aligned using FantaMorph Deluxe PRO (see Motta‐Mena & Scherf, 2017).

### Results

#### Set Intervals: Force Pressure

To explore whether click pressure was associated with participant confidence, a two-tailed Pearson’s correlation was calculated between average confidence ratings against average standardized change from baseline click pressure. The analysis revealed that for male faces, *r*(54) = .38, *p* < .01; female faces, *r*(54) = .55, *p* < .001; and merged faces, *r*(54) = .52, *p* < .001, click pressure was positively and significantly correlated with self-reports for response confidence. To explore whether click pressure was associated with gender uncertainty and task difficulty, a repeated measures analysis of variance (ANOVA) was conducted with independent variable Type of Face (male, female, merged) and dependent variable click-pressure responses. The analysis revealed that there were significant differences between different face types, *F*(1.74, 92.19) = 97.05, *p* < .001, partial eta-squared = .65, Greenhouse–Geisser corrected. Bonferroni-corrected pairwise comparisons revealed that merged faces (*M* = 0.09, *SD* = 0.02) were associated with lower click pressures than male (*M* = 0.15, *SD* = 0.05, *p* < .001, *d* = 1.58) and female faces (*M* = 0.13, *SD* = 0.05, *p* < .001, *d* = 1.05). Self-report confidence ratings also revealed the same pattern of results; merged faces (*M* = 3.43, *SD* = 0.56) were rated lower compared with male (*M* = 8.42, *SD* = 0.44, *p* < .001, *d* = 9.91) and female (*M* = 8.54, *SD* = 0.6, *p* < .001, *d* = 8.81) faces.

#### Set Intervals: Click-Release Time

To explore whether click-release time was associated with participant confidence, a two-tailed Pearson’s correlation was ran for click-release-time scores and confidence ratings. The analysis revealed that for male faces, *r* (54) = –.9, *p* < .001; female faces, *r*(54) = –.69, *p* < .001; and merged faces, *r*(54) = –.94, *p* < .001, click-release time was negatively and significantly correlated with self-reports for response confidence. To explore whether click-release time was associated with gender uncertainty and task difficulty, a repeated measures ANOVA was ran with independent variable Type of Face (male, female, merged) and dependent variable click-release-time responses. The analysis revealed that there were significant differences between face types, *F*(1.77, 93.89) = 83.76, *p* < .001, partial eta-squared = .61, Huynh-Feldt corrected. Bonferroni-corrected pairwise comparisons revealed that merged faces (*M* = 0.14, *SD* = 0.06) were associated with longer click-release times than male (*M* = 0.07, *SD* = 0.03, *p* < .001, *d* = –3.24) and female faces (*M* = 0.1, *SD* = 0.06, *p* < .001, *d* = –0.94). Intriguingly, the same pattern of results was not found in overall response time, in seconds, to the engagement task, *F*(2, 106) = 0.75, *p* = .48, partial eta-squared = .01. Bayesian analysis of pairwise comparisons (lower limit: –0.5, upper limit: 0.5) revealed that merged faces (*M* = 1.31, *SE* = 0.05) showed evidence for being significantly within the same intervals as male faces (*M* = 1.26, *SE* = 0.05, *B* = 0.17) and a trend for being within the same intervals as female faces (*M* = 1.23, *SE* = 0.05, *B* = 0.45) faces. These results suggest that click pressure and click-release time were associated with participant confidence and task difficulty in this part of the study. The findings also suggest that click-release time was a precise assessment of gender uncertainty and task.

#### Varying Intervals: Force Pressure and Click-Release Time

We wanted to explore whether these findings could be replicated in more complex experimental designs including set-of-steps variations of gender-related characteristics. To explore whether click pressure and click-release time were associated with participant confidence in set-of-steps variations of gender-related characteristics, a two-tailed Pearson’s correlation was ran. Click pressure was positively and significantly associated with participants’ confidence ratings, *r*(54) = .62, *p* < .001. Release time was negatively and significantly associated with participants’ confidence ratings, *r*(54) = –.63, *p* < .001. Detailed results can be seen in Table 1.

**Table 1.** Correlation Analysis for Force Pressure and Click-Release-Time Responses for Study One.

Male	Female	Confidence*M* (*SD*)	Force pressure*M* (*SD*)	*r*	*p* value (Bayes factor)	Release time*M* (*SD*)	*r*	*p* value
10%	90%	7.39 (0.59)	0.11 (0.04)	.36	<.01	0.18 (0.07)	– .87	<.001
30%	70%	5.76 (0.33)	0.06 (0.03)	.29	.03 (.63)	0.27 (0.09)	– .87	<.001
50%	50%	3.51 (0.77)	0.05 (0.03)	.01	.48 (4.63)	0.37 (0.08)	– .88	<.001
70%	30%	5.62 (0.31)	0.06 (0.03)	– .04	.73 (5.56)	0.28 (0.08)	– .88	<.001
90%	10%	7.43 (0.52)	0.11 (0.04)	.38	<.01	0.18 (0.08)	– .84	<.001

*Note*. Correlation coefficient Person’s *r* and significance values for confidence ratings and force pressure, and confidence ratings and click-release time for Study 1 for varying intervals of gender characteristics. For each nonsignificant result, a Bayes factor was calculated for the correlation analysis as the probability that these data would be observed if the null hypothesis were true (*p* (Data|H0); BF_01_ ≥ 10; Jarosz & Wiley, 2014). The Bayes factor was calculated as the likelihood ratio that these data would be observed if the null versus the alternative hypothesis were true (BF_01_). Following Dienes (2014), we considered BF_01_ ≥ 10 as evidence for the null.

To explore whether click pressure was associated with gender uncertainty and task difficulty, a repeated measures ANOVA was conducted with independent variable Gender Intervals (10%, 30%, 50%, 70%, 90%) and dependent variable force-pressure responses. The analysis revealed that there were significant differences between face types, *F*(1.79, 93.36) = 520.11, *p* < .001, partial eta-squared = .91, Greenhouse–Geisser corrected. The same pattern of results was reported for self-reports for confidence ratings, *F*(2.19, 116.34) = 684.94, *p* < .001, partial eta-squared = .93, Greenhouse–Geisser corrected. The same analysis was repeated for click-release-time responses. A repeated measures ANOVA with independent variables Gender Intervals and dependent variable click-release time revealed that there were very highly significant differences between different Gender Intervals, *F*(2.49, 131.84) = 1681.58, *p* < .001, partial eta-squared = .97, Greenhouse–Geisser corrected. Interestingly, in this stage, response time also revealed significant differences between Gender Intervals, *F*(3.2, 169.76) = 18.31, *p* < .001, partial eta-squared = .26, Greenhouse–Geisser corrected. This suggests that the variation in morphing characteristics had a very significant effect across all participant responses and resulted in very significant differences in confidence, response time, release time, and force pressure. Bonferroni-corrected pairwise comparisons for these effects can be seen in Table 2. These results suggest that force-pressure responses were associated with participant confidence in multiple set-of-steps gender morphing presentations. These findings also suggest that click-release-time responses were a very high and highly significant correlate of participant confidence and task difficulty in this stage of Study 1 (see also Supplementary Material 11.1 and 12.1).

**Table 2.** Comparisons for Click Pressure, Release and Response Time, and Confidence Ratings.

Male	Female	ConfidenceStandardized Cohen’s *d*	Response time*M* (*SD*) and standardized Cohen’s *d* (Bayes factor)	Force pressureStandardized Cohen’s *d*	Release timeStandardized Cohen’s *d*
10%	90%	2.62*	1.36 (0.36); –0.47 (0.64)	0.96*	–1.06*
30%	70%	–0.42*	1.44 (0.37); –0.13 (0.15)	–0.59*	0.14*
50%	50%	–3.75**	1.94 (0.62); 0.85**	–0.99**	1.43**
70%	30%	–0.76*	1.32 (0.37); –0.43 (1.23)	–0.54*	0.26*
90%	10%	2.87*	1.4 (0.41); –0.22 (0.26)	0.87*	–0.97*

*Note*. Standardized effect size Cohen’s *d* per assessment ((Condition Mean (*SD*) to (Σ_mean (_*_SD_*_)_ (intervals)/n (intervals))). Asterisk (*) indicates Bonferroni-corrected significance at *p* < .001 against all other scores in the same assessment (i.e., confidence, response time, force pressure, release time) except the opposite set-of-steps morphing condition (e.g., 30% male/70% female vs. 70%male/30% female). Double asterisks (**) indicate Bonferroni-corrected significance at *p* < .001 against all other scores in the same assessment. For each nonsignificant result, a Bayes factor (L.B.: –1; H.B: 1) was calculated for mean differences to the average condition mean (*M* = 1.49) and standard error (*SE* = 0.057) for response time to test evidence for the null (*B* < 0.33; Dienes, 2014).

## Study 2: Masked Stimuli

### Aims

The aim of the current study was to explore force-pad click-pressure and click-release-time responses to backwards masked emotional faces. We wanted to explore whether the current technology can be used to implicitly assess response confidence, task difficulty, and physiological arousal in masking studies. The hypothesis during this study was that, for set and varying durations of presentation (i.e., 27.78 ms and 13.89 or 20.83 or 27.78 or 34.72 or 41.67 ms), confidence in a response would be positively associated with click pressure and negatively associated with click-release time. In addition, we expected that shorter durations of presentation will result in higher task difficulty and lower confidence in responses and will be negatively associated with click pressure and positively associated with release time. We also hypothesized that force pressure could reveal physiological differences between different emotional types when using backwards masking, such as higher scores for emotional types that are characterized by high arousal (i.e., fearful faces) compared with other emotional types (i.e., sad and neutral faces). Finally, before every section, we ran a Bayesian analysis for subliminality. When the results suggested evidence for subliminal processing, further analysis of hit (true positive) and miss (false negative) responses was conducted for that duration of presentation.

### Participants

A power calculation based on medium effect sizes (partial eta-squared = .06, *f* = .25) was performed. The result revealed that 36 participants would be required for *P*_(1–β)_ ≥ .95 (Faul et al., 2009). A total of 56 volunteers (29 female) who were not part of Study 1 participated in the current study. The exclusion criteria and inclusion criteria were identical with Study 1. Data from two participants were excluded due to traits indicating a possible attention deficit hyperactivity disorder diagnosis. Data from four participants were excluded from the analysis due to movement-related artefacts (see Supplementary Material 7.1). Data from two participants were excluded due to scores (>94) that indicate possible alexithymic traits. The final population sample consisted of 48 (27 female) participants with mean age 28.81 (*SD* = 5.04). All participants were fully briefed concerning the physiological assessment after the experiment. The experiment was approved by the Ethics Committee of the School of Psychology of the University of Nottingham.

### Procedure

The experiment was presented on a high-frequency LED monitor set at 144 Hz (6.94 ms). To validate the presentation of brief backwards masked stimuli, an IPAD PRO camera with 240 Hz refresh rate (4.17 ms) recorded two pilot runs of the experiment, and the stimulus presentation was assessed frame by frame; no instances of dropped frames were detected. A self-developed dropped frame report script with one frame (6.94 ms) tolerance threshold was coded in Python, and two pilot experimental diagnostic sessions were run. The presenting monitor reported no dropped frames; prognostic dropped frame rate was estimated at 1/5,000 trials. Experimental stages were, subsequently, run using dropped frames diagnostics and per stimulus presentation frame rate performance of the stimuli presenting monitor; no instances of dropped frames were reported.

The facial stimuli used were taken from the dataset created by Gur et al. (2002). They included faces with fearful, sad, and neutral expressions. Nonfacial blurs were also generated from black and white pattern stimuli and scrambled using pseudorandomized pixel permutation in MATLAB. All the stimuli were adjusted for interpupillary distance, transformed to grey scale, and resized to a 1,024 × 768 pixels resolution. Their average luminance was controlled using the SHINE MATLAB toolbox. The faces were spatially aligned and placed in a white circle (height: 6 cm, width: 4 cm). The included stimuli were tested for physiological arousal during supraliminal presentations (1 s) and validated for emotional discrimination with FaceReader software (Noldus, 2018) and participant assessment; they were controlled for low-level visual features, such as spatial frequency and gradient orientation differences; and the black and white pattern mask was also separately compared and adjusted for luminance contrast with the presented faces (see Tsikandilakis & Chapman, 2018; Tsikandilakis et al., 2018, 2019a). 

### Experiments

Participants took part in a 5-min training stage. By the end of the training phase, they were asked whether they were ready to participate in the experiment. All participants replied positively. The current study included two stages with order randomized. Participants were allowed a 5-min break between each stage. During the engagement tasks of this study, all text was presented in black on a white background using a clearly visible and standard font (Times New Roman) and with a clearly visible font size (pt. 28). Each response-related part of text was boxed within a clearly visible and distinct selectable area with set dimensions (binary task: pt. 0.73 (H) to 2.33 (W); interval space: pt. 1.62 (W)). Participants were briefed during the training stage that they had the choice to restart the engagement task by pressing *space* in case of accidental miss-clicks; no instances of miss-clicks were reported.

**Figure 3.**
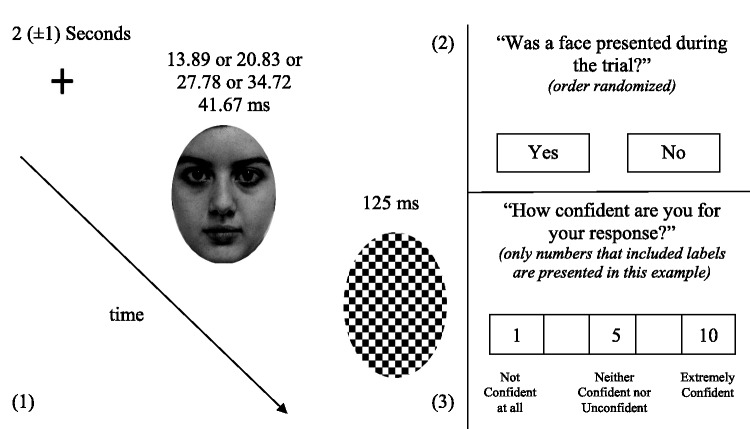
Experimental Sequence Study 2. In (1), participants watched masked stimuli for set (27.78 ms) and varying durations (13.89 or 20.83 or 27.78 or 34.72 or 41.67 ms). The stimuli were backward-masked with a black and white pattern mask (125 ms). They were afterwards assigned the engagement tasks illustrated in the figure: (2) and (3). Each engagement task was presented separately and in the described order. After each trial, a 7-s blank-screen interval was presented.

In one stage, participants were presented with a fixation cross for 2 (±1) s. After the fixation cross, a random fearful or sad or neutral face or a nonfacial blur was presented for 27.78 ms at fixation. A black and white pattern mask was then presented for 125 ms (see Figure 3). A blank interval screen was presented for 1 s. After the blank interval screen, participants were asked by an on-screen message to decide whether a face was presented (Yes/No) using the mouse. After this task, participants were asked from an on-screen message to rate the confidence for their response from 1 (*not confident at all*) to 10 (*extremely confident*) using the mouse. After each trial, a 7-s blank-screen interval was presented. During this stage, 20 fearful, sad, and neutral faces and 60 nonfacial blurs were presented.

In the other stage, the participants were presented with a fixation cross for 2 (±1) s. After the fixation cross, a random fearful or sad or neutral face or a nonfacial blur was presented for 13.89 or 20.83 or 27.78 or 34.72 or 41.67 ms. A black and white pattern mask was then presented for 125 ms (see Figure 3). A blank interval screen was presented for 1 s. After the blank interval screen, participants were asked by an on-screen message to decide whether a face was presented (Yes/No) using the mouse. After this task, participants were asked from an on-screen message to rate the confidence for their response from 1 (*not confident at all*) to 10 (*extremely confident*) using the mouse. After each trial, a 7-s blank-screen interval was presented. Four fearful, sad, and neutral faces were presented per duration (13.89 or 20.83 or 27.78 or 34.72 or 41.67 ms), and 60 nonfacial blurs were presented during this stage. No actor identity was repeated during the study.

### Results and Discussion: Set Intervals: Subliminality

**Figure 4.**
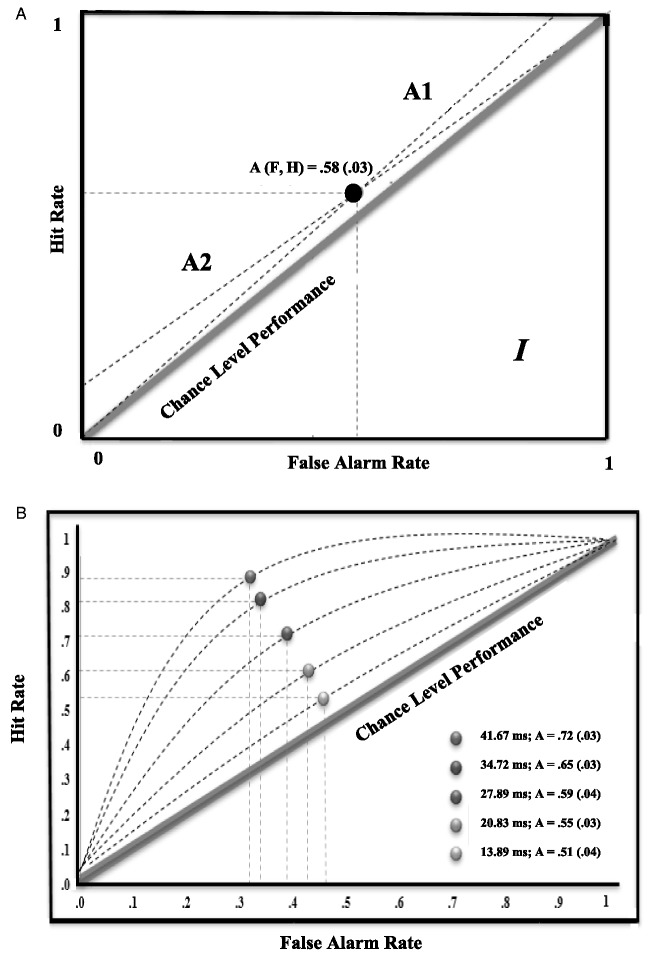
Receiver Operating Characteristics for Set (A) and Varying (B) Intervals for Backwards Masked Faces. In A, detection performance mean (*SD*) for 27.78 ms arranged according to a single threshold design including A1 and A2 intervals for possible range of varying (F, H) characteristics for detection performance (Zhang & Mueller, 2005). In B, detection performance for varying intervals arranged according to a multiple thresholds design (Fawcett, 2006).

Detection performance for stimuli presented for 27.78 ms was transformed to nonparametric sensitivity index *A* (Zhang & Mueller, 2005). A Bayesian analysis, uncorrected for degrees of freedom (Berry & Stangl, 2018), was run using the Dienes (2016) calculator to assess chance-level processing, with substantial evidence for the null hypothesis defined as a Bayes factor *B* below 1/3 (chance-level performance) and evidence for the alternate defined as a Bayes factor *B* above 3 (different to chance-level performance). The intervals were defined at –0.1 (0.4; lower bound [L.B.]) and 1 (0.6; higher bound [H.B.]), with 0 (*A* = 0.5) representing chance-level performance. Detection performance using nonparametric receiver operating characteristics was overall above chance (*M* = 0.5843, *SE* = 0.0071, *B* > 3) suggesting that faces presented for 27.78 ms were not processed subliminally (see Figure 4).

### Results and Discussion: Set Interval: Force Pressure

To explore whether click pressure was associated with participant confidence in response to briefly presented backwards masked faces, a two-tailed Pearson’s correlation was ran for click-pressure scores and confidence ratings. The analysis revealed that click pressure was positively and significantly correlated with self-reports for response confidence, *r*(48) = .6, *p* < .001. A similar pattern of results was reported for fearful, *r*(48) = .32, *p* = .027; sad, *r*(48) = .63, *p* < .001; and neutral, *r*(48) = .57, *p* < .001, faces. To explore whether force pressure was associated with differences relating to physiological responses to the presented emotion, a repeated measures ANOVA was ran with independent variables Type of Face (fearful, sad, neutral) and dependent variable force pressure. The analysis revealed that there were significant differences between face types, *F*(1.36, 63.97) = 179.03, *p* < .001, partial eta-squared = .79, Huynh-Feldt corrected. Bonferroni-corrected pairwise comparisons revealed that fearful faces (*M* = 0.27, *SD* = 0.09) elicited higher force pressure than sad (*M* = 0.12, *SD* = 0.04, *p* < .001, *d* = 2.15) and neutral faces (*M* = 0.1, *SD* = 0.06, *p* < .001, *d* = 2.22). Sad (*SE* = 0.005) and neutral (*SE* = 0.005) faces revealed substantial evidence for being within the same intervals for force pressure (L.B.: –0.05, H.B.: 0.05, *B* = 0.17).

### Results and Discussion: Set Interval: Click-Release Time

To explore whether click-release time was associated with participant confidence in response to briefly presented backwards masked faces (27.78 ms), a two-tailed Pearson’s correlation was ran for click-release-time scores and confidence ratings. The analysis revealed that click-release time was negatively and highly significantly correlated with self-reports for response confidence, *r*(48) = –.72, *p* < .001. The same effect was revealed for fearful, *r*(48) *=* –.48, *p* < .001; sad, *r*(48) *=* –.41, *p* < .01; and neutral, *r*(48) *=* –.71, *p* < .001, faces. These findings suggest that release time was associated with participant confidence.

### Results and Discussion: Varying Intervals: Subliminality

Detection performance per duration of presentation (13.89, 20.83, 27.78, 34.72, and 41.67 ms) was transformed to nonparametric sensitivity index *A* (Zhang & Mueller, 2005). A Bayesian analysis, uncorrected for degrees of freedom, was run using the Dienes (2016) calculator to assess chance-level processing, with substantial evidence for the null hypothesis defined as a Bayes factor *B* below 1/3 (chance-level performance) and evidence for the alternate defined as a Bayes factor *B* above 3 (different to chance-level perfoance). The intervals were defined at –0.1 (0.4; L.B.) and 0.1 (0.6; H.B.), with 0 (*A* = 0.5) representing chance-level performance. Detection performance using nonparametric receiver operating characteristics was overall above chance for faces presented for 41.67 ms (*M* = 0.7185, *SD* = 0.0256, *SE* = 0.0037, *B* > 3), 34.72 ms (*M* = 0.6519, *SD* = 0.0284, *SE* = 0.0041, *B* > 3), 27.78 ms (*M* = 0.5928, *SD* = 0.0353, *SE* = 0.0051, *B* > 3), and 20.83 ms (*M* = 0.5457, *SD* = 0.0290, *SE* = 0.0042, *B* > 3). Faces presented for 13.89 ms revealed a trend (*M* = 0.5113, *SD* = 0.0436, *SE* = 0.0063, *B* = 0.39) for chance-level performance (see Figure 4).

### Results and Discussion: Varying Intervals: Force Pressure and Click-Release Time

We wanted to explore whether these findings could be replicated in more complex experimental designs including set-of-steps variations of backwards masked faces. To explore whether click pressure and click-release time were associated with participant confidence in set-of-steps variations of backwards masked faces, a two-tailed Pearson’s correlation was ran. Click pressure was positively and significantly associated with participant confidence, *r*(48) = .92, *p* < .001. Release time was negatively and significantly associated with participants confidence, *r*(48) = –.83, *p* < .001. Detailed results can be seen in Table 3.

**Table 3.** Correlation Analysis for Force Pressure and Click-Release-Time Responses for Study Two.

Time	Type	Confidence*M* (*SD*)	Force pressure*M* (*SD*)	*r*	*p* value (Bayes factor)	Release time*M* (*SD*)	*r*	*p* value (Bayes factor)
21 ms	Fear	4.61 (0.79)	0.21 (0.02)	.49	<.001	0.36 (0.07)	–.82	<.001
	Sad	4.36 (1.03)	0.11 (0.01)	.46	<.001	0.36 (0.07)	–.73	<.001
	Neutral	4.56 (0.9)	0.11 (0.01)	.16	.28 (3.13)	0.39 (0.08)	–.04	.29 (3.26)
	Overall	4.51 (0.62)	0.14 (0.01)	.67	<.001	0.37 (0.07)	–.73	<.001
28 ms	Fear	6.14 (0.63)	0.25 (0.04)	.44	.002	0.29 (0.07)	–.17	.27 (3.02)
	Sad	5.95 (0.68)	0.13 (0.02)	.65	<.001	0.27 (0.07)	–.36	.01
	Neutral	6.12 (0.65)	0.12 (0.02)	.51	<.001	0.27 (0.07)	–.95	<.001
	Overall	6.07 (0.59)	0.17 (0.02)	.68	<.001	0.28 (0.05)	–.73	<.001
35 ms	Fear	6.70 (0.47)	0.28 (0.06)	.36	.01	0.28 (0.07)	–.54	<.001
	Sad	6.83 (0.43)	0.15 (0.03)	.07	.65 (4.97)	0.27 (0.07)	.17	.24 (2.84)
	Neutral	6.76 (0.47)	0.15 (0.03)	.12	.43 (4.08)	0.25 (0.08)	–.46	<.001
	Overall	6.76 (0.33)	0.19 (0.03)	.4	.005	0.27 (0.04)	–.59	<.001
42 ms	Fear	6.91 (0.56)	0.34 (0.03)	.66	<.001	0.27 (0.07)	–.75	<.001
	Sad	6.88 (0.58)	0.15 (0.03)	.09	.55 (4.62)	0.28 (0.08)	.09	.51 (4.45)
	Neutral	7.11 (0.54)	0.15 (0.03)	.56	<.001	0.28 (0.07)	.14	.34 (3.56)
	Overall	6.97 (0.33)	0.21 (0.03)	.69	<.001	0.28 (0.07)	–.54	<.001

*Note*. Correlation coefficient Person’s *r* and significance values for confidence ratings and force pressure, and confidence ratings and click-release time for Study 2 for varying intervals of backwards masked faces as described in the left of the current table. For each nonsignificant result, a Bayes factor was calculated for the correlation analysis as the probability that these data would be observed if the null hypothesis were true (*p* (Data|H0); BF_01_ ≥ 10). The Bayes factor was calculated as the likelihood ratio that these data would be observed if the null versus the alternative hypothesis were true (BF_01_). We considered BF_01_ ≥ 10 as evidence for the null. Statistics and analysis for 13.89 ms are presented in a separate section.

To explore whether click pressure was associated with uncertainty and task difficulty, a repeated measures ANOVA was ran with independent variables Duration Intervals (20.83, 27.78, 34.72, and 41.67 ms) and Type of Emotion (Fear, Sad and Neutral) with dependent variable force-pressure responses. The analysis revealed that there were significant differences between Duration Intervals, *F*(2.45, 112.57) = 176.35, *p* < .001, partial eta-squared = .79, Greenhouse–Geisser corrected; Types of Emotion, *F*(1.44, 66.53) = 952.42, *p* < .001, partial eta-squared = .96, Greenhouse–Geisser corrected; and a significant interaction, *F*(3.24, 149.01) = 26.45, *p* < .001, partial eta-squared = .37, Greenhouse–Geisser corrected. A similar pattern of results was reported for self-reports for confidence ratings for Durations Intervals only, *F*(2.09, 96.26) = 499.98, *p* < .001, partial eta-squared = .92, Huynh-Feldt corrected. The same analysis was repeated for click-release-time responses. A repeated measures ANOVA with independent variables Duration Intervals and Type of Emotion and dependent variable click-release time revealed that there were significant differences between different durations, *F*(2.57, 118.27) = 97.59, *p* < .001, partial eta-squared = .68, Greenhouse–Geisser corrected. The analysis for response time did not reveal significant differences and revealed substantial evidence for response times being within the same intervals (L.B: –0.5, H.B: 0.5) between different durations, *F*(3, 138) = 1.41, *p* = .23, partial eta-squared = .03, *B* = 0.17. Bonferroni-corrected pairwise comparisons for these effects can be seen in Table 4. These results suggest that force-pressure responses were a potentially accurate assessment of participant confidence and physiological responses to brief backwards masked faces. These findings also suggest that click-release-time responses were a significant correlate of participant confidence and task difficulty in this study (see also Supplementary Material 11.2 and 12.2).

**Table 4.** Comparison Matrix for Click Pressure, Click-Release Time, and Confidence Ratings.

A. Comparison matrix per emotional type												
Time	Type			ConfidenceCohen’s *d* (Bayes factor)					Force pressureCohen’s *d* (Bayes factor)		Release timeCohen’s *d* (Bayes factor)	
				Sad		Neutral			Sad	Neutral	Sad	Neutral
21 ms	Fear			0.28 (0.22)		0.09 (0.01)			5.45*	5.73*	–0.09 (0.01)	–0.37*
	Sad					–0.22 (0.19)				0.15 (0.01)		–0.28 (0.23)
28 ms	Fear			0.29 (0.23)		0.01 (0.01)			3.89*	4.05*	0.05 (0.01)	0.04 (0.01)
	Sad					–0.26 (0.22)				0.22 (0.02)		–0.02 (0.01)
35 ms	Fear			–0.31 (0.29)		–0.15 (0.12)			2.93*	2.78*	0.17 (0.14)	0.44**
	Sad					0.16 (0.12)				–0.11 (0.01)		0.26 (0.21)
42 ms	Fear			0.07 (0.01)		–0.36*			3.46*	3.61*	0.14 (0.12)	0.25 (0.21)
	Sad					–0.43*				0.19 (0.05)		–0.09 (0.01)
Total	Fear			0.24 (0.21)		–0.12 (0.14)			3.93*	4.04*	0.07 (0.01)	–0.26 (0.23)
	Sad					–0.19 (0.17)				0.11 (0.14)		0.05 (0.01)
B. Comparisons matrix per duration of presentation												
		28 msCohen’s *d* (Bayes factor)					35 msCohen’s *d* (Bayes factor)			42 msCohen’s *d* (Bayes factor)		
		Conf.	F.P.		R.T.		Conf.	F.P.	R.T.	Conf.	F.P.	R.T.
21ms		–2.51*	–1.48*		1.46*		–4.53*	–2.01*	1.76**	–4.93**	–3.03**	1.63**
28 ms							–1.48*	–0.98*	0.25 (0.21)	–1.93*	–1.81**	0.07 (0.01)
35 ms										–0.63*	–0.6*	0.28 (0.23)

*Note*. In A, Cohen’s *d* emotional type and assessment. In B, Bonferroni-corrected comparisons for confidence (Conf.), force pressure (F.P.), and click-release time (R.T.) with Cohen’s *d* scores between different durations. Asterisk (*) indicates Bonferroni-corrected significance at *p* < .001 in comparison with other items in the same duration. For each nonsignificant result, a Bayes factor (L.B.: –0.05 (force pressure) or –0.1 (release time) or –1 (confidence responses); H.B: 0.5 or 0.1 or 1) was calculated for mean differences to the average condition mean and standard error for that condition (see Table 3) to test evidence for the null (*B* < 0.33; Dienes, 2014, 2016). Statistics and analysis for 13.89 ms are presented in a separate section.

### Results and Discussion: Confidence, Click Pressure, Release Time, and Subliminality

Signal detection analysis for faces presented for 13.89 ms revealed a trend for Bayesian evidence for the null—that detection performance was within a priori defined criteria for chance-level performance—suggesting that these stimuli could have been processed subliminally (*M* = 0.5113, *SD* = 0.0436, *SE* = 0.0063, *B* = 0.39). To explore responses to subliminal emotional faces and also whether the current technology could be used for the physiological assessment of subliminal emotion, we used the analysis model for the assessment of subliminality described in previous publications (Tsikandilakis & Chapman, 2018; Tsikandilakis et al., 2018, 2019b, 2019c, 2019d; Tsikandilakis, Kausel, et al., 2019). To explore whether participants responded to subliminal faces, a repeated measures ANOVA with independent variables Type of Emotion (Fear, Sadness, Neutral) and Detection Response (Hits, Misses) was ran for confidence rating, click-release time, and force pressure responses. The analysis revealed no significant differences and also revealed strong evidence for the null for Type of Emotion for release-time responses, *F*(2, 92) = 0.051, *p* = .95, partial eta-squared < .01, *B* = 0.03, and confidence rating self-reports, *F*(2, 92) = 0.181, *p* = .84, partial eta-squared < .01, *B* = 0.11. The analysis also revealed no significant differences and revealed evidence for the null for Detection Responses and a Type of Emotion to Detection Response interaction for release time (*p*_detection_ = .89, *B* = 0.06, *p*_interaction_ = .87, *B* = 0.08) and confidence self-reports (*p*_detection_ = .93, *B* = 0.05, *p*_interaction_ = .65, *B* = 0.41).

**Figure 5.**
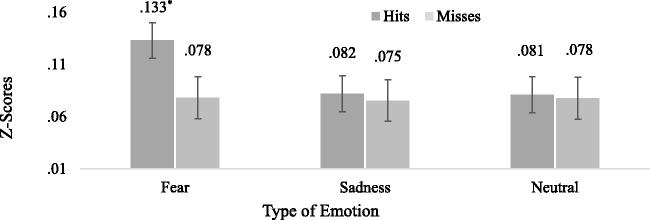
Hit and Miss Responses for Force Pressure. Analysis for hits and miss responses for force pressure for faces presented for 13.89 ms. Asterisk (*) indicates Bonferroni-corrected significance at *p* < .001.

A different pattern of results was revealed for force-pressure responses. Significant differences were revealed for Type of Emotion, *F*(1.58, 72.75) = 68.83, *p* < .001, partial eta-squared = .59, Greenhouse–Geisser corrected, and Detection Performance, *F*(1, 46) = 59.93, *p* < .001, partial eta-squared = .57, and significance was also revealed for a Type of Emotion to Detection Performance interaction, *F*(1.29, 59.47) = 52.78, *p* < .001, partial eta-squared = .53, Huynh-Feldt corrected. Bonferroni-corrected pairwise comparisons revealed that hits for fearful faces (*M* = 0.13, *SD* = 0.02) were higher for force-pressure responses than hits for sad (*M* = 0.08, *SD* = 0.01, *p* < .001, *d* = 3.16) and neutral faces (*M* = 0.08, *SD* = 0.01, *p* < .001, *d* = 3.06). On the contrary, hit responses between sad and neutral faces (*p* = .71, *d* = 0.04, *B* = 0.23), and miss responses between fearful faces (*M* = 0.08, *SD* = 0.04) and miss responses for sad (*M* = 0.08, *SD* = 0.01, *p* = .97, *d* = 0.01, *B* = 0.03) and neutral faces (*M* = 0.08, *SD* = 0.01, *p* = .67, *d* = 0.03, *B* = 0.19) were not significantly different and revealed evidence for the null (*B* < 0.33). A similar pattern of results was revealed between miss responses for sad and neutral faces (*p* = .38, *d* = 0.17, *B* = 0.1). These results suggest that faces presented for 13.89 ms did not provide evidence for subliminal processing (see also Figure 5).

The higher scores for force pressure reported in response to fearful faces compared with other facial stimuli in this study could suggest either that, as we have shown repeatedly in our previous research, fearful faces are due to biological (Brooks et al., 2012) or salience-related (van der Ploeg et al., 2017) reasons more discernible during backwards masking resulting in higher confidence for detection (Tsikandilakis & Chapman, 2018; Tsikandilakis et al., 2018) or that the current device could also be suited for the implicit exploration of physiological arousal in response to emotional elicitors (see, e.g., Tsikandilakis et al., 2019c; see also Supplementary Material 6.1).

## Study 3: Target Search

### Aims

The aim of the current study was to explore force-pad click pressure and click-release-time responses in a letter-search task. We also test whether the current technology has application in studies that do not include facial stimuli. The hypotheses during this stage were that in set search for letter strings, higher participant confidence will be positively associated with higher click pressure and negatively associated with release time. Another set of hypotheses during this stage was that for varying string lengths during the search task (i.e., two- or three- or four-letter strings), longer letter strings length would result in higher task difficulty and response uncertainty and, therefore, that longer search letter strings will be associated with lower click pressure and higher click-release-time responses.

### Participants

A power calculation based on medium effect sizes (partial eta-squared = .06, *f* = .25) was performed. The result revealed that 43 participants would be required for *P*_(1–β)_ ≥ .95 (Faul et al., 2009). A total of 66 volunteers (34 female) who were not part of Studies 1 and 2 participated in the current study. The exclusion and inclusion criteria included those implemented during Studies 1 and 2 and diagnosis for dyslexia or a reading disorder through self-report. The participants were screened with the Adult Reading History Questionnaire (Lefly & Pennington, 2000), and participants with scores above 0.3 were excluded from the analysis; data from two participants were excluded. Data from three participants were also excluded due to reporting scores that indicated a possible psychiatric diagnosis. One participant was excluded from the analysis due to movement-related artifacts (see Supplementary Material 7.1). The final population sample consisted of 69 (32 female) participants with mean age 31.68 (*SD* = 7.81). The participants were native (26) and nonnative (34) English speakers (see Supplementary Material 5.1). All participants were fully briefed concerning the physiological assessment after the experiment. The experiment was approved by the Ethics Committee of the School of Psychology of the University of Nottingham.

### Experiments

Participants took part in a 5-min training stage. By the end of the training phase, they were asked whether they were ready to participate in the experiment. All participants replied positively. In the main experiment, the letters were presented in black colour on a white background using Times New Roman, font size 28. All letter stimuli were presented at fixation. The current study included two stages with order randomized. Participants were allowed a 5-min break between each stage. During the engagement tasks in this study, all text was presented in black on a white background using a clearly visible and standard font (Times New Roman), with a clearly visible font size (pt. 28). Each response-related part of text was boxed within a clearly visible and distinct selectable area with set dimensions (binary task: pt. 0.73 (H) to 2.33 (W); interval space: pt. 1.62 (W)). Participants were briefed during the training stage that they had the choice to restart the engagement task by pressing *space* in case of accidental miss-clicks; no instances of miss-clicks were reported.

In one stage, participants were presented with a fixation cross for 2 (±1) s. After the fixation cross, a random target letter was presented for 125 ms. A black and white pattern mask (pt. 1.34 (H) to 2.26 (W)) was then presented for 125 ms. After the mask, a blank interval screen was presented for 1 s. After the blank interval screen, a random three-letter string was presented for 125 ms. A black and white pattern mask (pt. 1.34 (H) to 2.26 (W)) was then presented for 125 ms. After the mask, a blank interval screen was presented for 1 s. After the blank interval screen, participants were asked by an on-screen message to decide whether the target letter was present in the letter string (Yes/No) using the mouse. After this task, participants were asked from an on-screen message to rate the confidence for their response from 1 (*not confident at all*) to 10 (*extremely confident*) using the mouse. After each trial, a 7-s blank-screen interval was presented. A total of 52 pseudorandomized letters were presented during this experiment. A total of 26-letter strings including the target letter (position randomized) were presented, and a total of 26 random letter strings not including the random letter were presented (Figure 4). Every letter was presented once for the target including condition and once for nontarget including condition. After each trial, the presented target letter was disqualified from inclusion in the next letter-search task. Instances of repeated randomly generated letter strings were not reported in within-participant trials. The set letter strings were examined postexperimentally for the formulation of meaningful and identifiable words; instances of pronounceable pseudowords were reported (i.e., AVE, HAN, HIL, PTO); instances of random real-word formation were recorded (i.e., APE, ARE, ATE, BUY, DOS,^2^ DRY, FIN, PAY, MAY, SAY, SIT, SAT, TOP). These instances (*n* = 20; see the following paragraph) were removed from the analysis.

**Figure 6.**
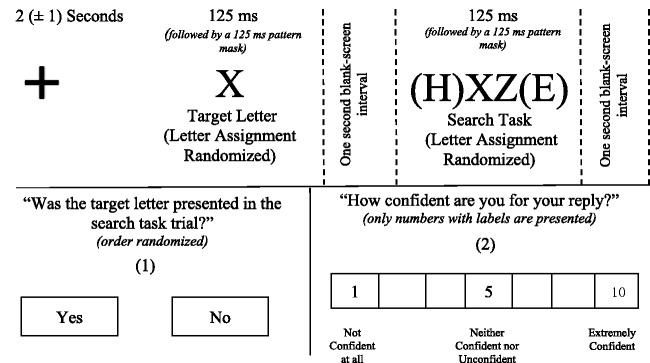
Example Experimental Sequence Study 3. Participants were asked to perform a letter-search task in a set (three-letter string) and varying in complexity target (two- or three- or four-letter string). They were afterwards assigned the engagement tasks illustrated in the figure: (1) and (2). Each engagement task was presented separately and in the described order. No text was included in the presentation. The participants were instructed in the training phase concerning the target letter and search task episodes included in the experiment. All participants responded positively that they could understand and undertake the task. After each trial, a 7-s blank-screen interval was presented before the next experimental sequence.

In the other stage, participants were presented with a fixation cross for 2 (±1) s. After the fixation cross, a random letter was presented for 125 ms. A black and white pattern mask (pt. 1.34 (H) to 2.26 (W)) was then presented for 125 ms. After the mask, a blank interval screen was presented for 1 s. After the blank interval screen, a two- or three- or four-letter string was presented for 125 ms. A black and white pattern mask (pt. 1.34 (H) to 2.26 (W)) was then presented for 125 ms. After the mask, a blank interval screen was presented for 1 s. After the blank interval screen, participants were asked by an on-screen message to decide whether the target was presented in the string (Yes/No) using the mouse. After this task, participants were asked from an on-screen message to rate the confidence for their response from 1 (*not confident at all*) to 10 (*extremely confident*) using the mouse. After each trial, a 7-s blank-screen interval was presented. A total of 52 pseudorandomized letters were presented during this experiment. A total of 11-letter strings per string length (two or three or four) including the target letter were presented, and a total of 11 letters strings per string length not including the target letter were presented (Figure 6). A target letter was presented once for the target including and once for nontarget including conditions per string length. After each trial, the presented target letter was disqualified from inclusion in the next letter-search task. The letter strings were examined postexperimentally. Instances of repeated randomly generated letter strings were reported in within-participants between-stages trials (i.e., AHL, BRT, JFO), and these were excluded. Instances of pseudowords and random real-word formations that were within-participants present in a previous stage were excluded (i.e., ARE, AVE, PAY, PTO); instances of pronounceable pseudowords were reported (i.e., DEI, GHART, GLON, HA, PHON, YA, YO); instances of random real-word formation were reported (i.e., AIM, EXIT. EDIT, GO, GOD, HAM, HARM, HEY, HI, SAY, SIR, SO, YES). These instances (*n* = 27) were removed from the analysis.

### Results and Discussion Set Intervals: Force Pressure and Release Time

To explore whether click pressure was associated with participant confidence, a two-tailed Pearson’s correlation was calculated between click pressure scores and confidence ratings. The analysis revealed that click pressure was positively and significantly correlated with self-reports for response confidence, *r*(60) *=* .6, *p* < .001. A significant association was also reported for click-release time and confidence ratings. Click-release time was significantly and negatively associated with confidence ratings, *r*(60) = –.47, *p* < .001. These results suggest that click pressure and click release-time were associated with participant confidence and task difficulty in this part of the study.

### Results: Varying Intervals: Force Pressure and Click-Release Time

We wanted to explore whether these findings could be replicated in more complex experimental designs including set-of-steps variations of letter strings. To explore whether click pressure and click-release time were associated with participant confidence in set-of-steps variations of letter strings, a two-tailed Pearson’s correlations was calculated. Click pressure was positively and significantly associated with participant confidence for two, *r*(60) = .57, *p* < .001, and three, *r*(60) = .81, *p* < .001, but not four-letter string intervals, *r*(60) = .04, *p* = .77, *B*_01_ = .17. Release time was negatively and significantly associated with participants confidence for two-, *r*(60) = –.36, *p* < .01; three-, *r*(60) = –.87, *p* < .001; and four-letter string intervals, *r*(60) = –.57, *p* < .001.

To explore whether click pressure was associated with task difficulty, a repeated measures ANOVA was conducted with independent variable Letter-String Intervals (two, three, four) and dependent variable force-pressure responses. The analysis revealed that there were significant differences between different string intervals, *F*(2, 118) = 69.99, *p* < .001, partial eta-squared = .54. The same pattern of results was reported for self-reports for confidence ratings, *F*(2, 118) = 168.04, *p* < .001, partial eta-squared = .74. The same analysis was repeated for click-release-time responses. A repeated measures ANOVA with independent variables Letter-String Intervals and dependent variable click-release time revealed that there were significant differences between different intervals, *F*(1.72, 101.51) = 348.49, *p* < .001, partial eta-squared = .86; Greenhouse–Geisser corrected. Response time also revealed significant differences between letter intervals, *F*(2, 118) = 5.16, *p* < .01, partial eta-squared = .08. Bonferroni-corrected pairwise comparisons for these effects can be seen in Table 5. These results suggest that force-pressure responses were associated with participant confidence in this stage. These findings also suggest that click-release-time responses were a significant correlate of participant confidence and task difficulty in this stage of Study 3 (see also Supplementary Material 11.3 and 12.3).

**Table 5.** Participant Responses for Study 3 Varying Intervals.

A. Descriptive statistics for Study 3 varying intervals				
	Confidence*M* (*SD*)	Response time*M* (*SD*)	Release time*M* (*SD*)	Force pressure*M* (*SD*)
Two-letter string	6.62 (0.89)	1.51 (0.23)	0.38 (0.09)	0.13 (0.04)
Three-letter string	5.75 (1.11)	1.53 (0.22)	0.5 (0.12)	0.11 (0.03)
Four-letter string	3.89 (1.14)	1.63 (0.24)	0.69 (0.19)	0.07 (0.03)
B. Effect size Cohen’s *d* for Bonferroni-corrected comparisons				
	Confidence (Bayes factor)		Response time (Bayes factor)	
	Three-letter	Four-letter	Three-letter	Four-letter
Two-letter	0.88*	2.72*	–0.09 (0.05)	–0.55*
Three-letter		1.65*		–0.43*
	Release time (Bayes factor)		Force pressure (Bayes factor)	
	Three-letter	Four-letter	Three-letter	Four-letter
Two-letter	–1.38*	–2.11*	0.54*	1.78*
Three-letter		–1.23*		1.39*

*Note*. In A, mean and standard deviation per participant response. In B, effect size Cohen’s *d* and significance for Bonferroni-corrected pairwise comparisons. Asterisk (*) indicates Bonferroni-corrected significance at *p* < .001. For each nonsignificant result, a Bayes factor (L.B.: –0.05 (force pressure) or –0.1 (release time) or –1 (confidence responses and response time); H.B: 0.5 or 0.1 or 1) was to test evidence for the null (*B* < 0.33; Dienes, 2014, 2016).

## Discussion

In the current studies, we tested the first, to our knowledge, application of a force-pressure assessment device disguised as a mouse pad in psychological studies. The device was successfully implemented in all three studies implicitly. Three out of 163 participants reported that their responses could be monitored in some way and reported that this monitoring was made via camera recording (see Supplementary Material 4.1). In the current studies, force pressure was negatively associated with task difficulty and positively associated with response confidence and revealed very high effect sizes for being able to measure these characteristics. During Experiments 1, 2, and 3, click-release times were associated more highly with task difficulty than reaction times were, suggesting that click-release time could be a useful alternative measure of human performance. As we have shown in a previous publication where we explicitly implemented an earlier version of the current device, there were evidence for differences in force pressure to very briefly presented fearful faces possibly compared with other stimuli types (Tsikandilakis et al., 2019c). These differences could be due to fearful faces being more salient and discernible and/or because the current equipment correlates with measures of physiological arousal such as skin conductance and heart rate (van der Ploeg et al., 2017). The results in the current article also suggest that when the device was implemented implicitly, the analysis did not provide evidence for subliminal processing during Study 2 because all recorded physiological responses were driven by correct detection of a presented emotional face (see also Pessoa & Adolphs, 2010).

Previous research has suggested that participants could use self-presentation strategies for responding to explicit assessment tasks. These can include self-presentation in a way that is perceived positive on a personal, interpersonal, and professional level (Strack & Deutsch, 2004). Additional research has suggested that certain participants overstate the task difficulty of an engagement task and downrate their response confidence for their replies due to conservative strategies for self-presentation, such as presenting oneself as attempting to perform at the best of their ability despite low performance. Conversely, previous research has suggested that certain participants consistently downrate task difficulty and overstate their response confidence to come across as overachievers (Hellmann, 2016; Kosakowska-Berezecka et al., 2017).

Previous research has attempted to explore these effects using implicit assessment of task difficulty and response confidence and reported very promising and remarkably high effects sizes for this assessment (see, e.g., Kaklauskas et al., 2009). This line of research, therefore, is a very promising area relating to psychological assessment. In the current studies, we found that lower force-pressure responses and higher click-release times were consistently associated throughout all studies with higher difficulty. Reduced difficulty was associated with higher force pressure and lower click-release times. We found that the current technology can be used to assess responses relating to task difficulty and participant confidence in psychological experiments, such as morphing, gender recognition, masking, and letter-search studies.

In previous studies, other researchers found highly significant findings and very high correlation coefficient effect sizes when assessing responses in experimental designs that were similar to the current studies (see, e.g., Campanella et al., 2001, p. 11; *F* = 3680; but see also particularly LeBel & Paunonen, 2011). In the current studies, we were also able to report highly significant results and correlation coefficients for force pressure and task difficulty and response confidence. Intriguingly, nevertheless, in a previous explicit assessment of force-pressure responses using a prototype of the current device, we found relatively smaller effect sizes for force-pressure responses to masked emotional faces (Tsikandilakis et al., 2019c, p. 7; *F* = 375.38). Because a firmly conclusive model concerning whether implicit and explicit physiological responses differ in relation to neural processing is wanting in the current area (Pessoa & Adolphs, 2010; Stanley et al., 2008), it is worth raising here the issue of whether implicit assessment of responses to engagement tasks that proceed particularly emotional presentations involve different neural systems to explicit assessments, such as possibly limbic system-related neural structures (Braunstein et al., 2017; Brooks et al., 2012; but see also Tsikandilakis et al., 2019b). This possibility could provide support for the argument that implicit responses are less regulated by inhibitory mechanisms and that they are possibly more revealing indicators of participant feedback when compared with explicit assessments (Strack & Deutsch, 2004).

A very promising finding in the current studies was that click-press-to-release times were a precise estimate of task difficulty (Colonius & Diederich, 2017). This effect is novel and an exploratory interpretation of why release times but not response times provided significant differences in the current experiments could relate to the idea that participants are conditioned to replying to engagement tasks within a specific time frame due to experience with experimental procedures. Another interpretation could relate to the idea that conflict and reflection in response to an engagement task inquiry could be explicitly assigned a specific, fixed, and finite time window for resolution in standard engagement tasks (see, e.g., Evans et al., 2015). This effect could not apply to click-press-to-release time because this could be an implicit confidence-related behavioural outcome that is not subject to equally well-structured time monitoring response strategies (Strack & Deutsch, 2004).

A very important final consideration that we should also raise relating to the key finding mentioned earlier is that click-press-to-release time can be accurately monitored using the current device but does not per se require the current device. For example, an intermediate in complexity manual coding function is included in the Supplementary Material of the current article, written by the current authors, that is designed to monitor click-press-to-release time for mouse responses (Supplementary Material 9.1) and keyboard components (Supplementary Material 9.2) using standard mouse equipment. This renders experimentation and further exploration more accessible by further research. This is an important aspect of the current research, and it is possible that the more seminal finding of the current study was that click-press-to-release times were an accurate measure of human performance. This is a potentially seminal finding. In addition to these, we should of course acknowledge and think further what other applications (Allgöwer et al., 2017), device formats, (Perkins et al., 2009), assessment measures (Bradley & Lang, 2000), and force pressure technology can be used for in relation to psychological research and research in general (Baumeister et al., 2007).

## Conclusions

In the current article, we presented the first, to our knowledge, force-pressure measuring device disguised as a mouse pad for the assessment of task difficulty and response confidence in psychological studies. We tested the device in three experimental designs. The studies comprised of a gender-recognition study using morphed male and female faces, a visual suppression study using backwards masking and a letter-search study that included deciding whether a target letter was repeated in a subsequently presented letter string. Across all studies, higher task difficulty was associated with higher click-release-time responses. Higher task difficulty was, intriguingly, also associated with lower click pressure. Higher confidence ratings were consistently associated with higher click pressure and shorter click-release time across all experiments. These findings suggest that the current technology can be used to assess responses relating to task difficulty and participant confidence. We also suggest that the assessment of release times could be implemented using simple code components, and we provide manual and easy-to-use code for the implementation.

## Supplemental Material

sj-pdf-1-ipe-10.1177_2041669520913319 - Supplemental material for “The Harder One Tries …”: Findings and Insights From the Application of Covert Response Pressure Assessment Technology in Three Studies of Visual Perception Click here for additional data file. Supplemental material, sj-pdf-1-ipe-10.1177_2041669520913319 for “The Harder One Tries …”: Findings and Insights From the Application of Covert Response Pressure Assessment Technology in Three Studies of Visual Perception by Myron Tsikandilakis, Persefoni Bali, Giannis Haralabopoulos, Jan Derrfuss and Peter Chapman in i-Perception

## Notes

1 Minimum and maximum (range) recorded pressure for a click response (see Supplemental Material 7.1).

2 An acronym for disk operating system.

## References

[bibr1-2041669520913319] Akamatsu M. MacKenzie I. S. (1996). Movement characteristics using a mouse with tactile and force feedback. International Journal of Human-Computer Studies, 45(4), 483–493.

[bibr2-2041669520913319] Akamatsu M. Sato S. (1994). A multi-modal mouse with tactile and force feedback. International Journal of Human-Computer Studies, 40(3), 443–453.

[bibr3-2041669520913319] Alexithymia. (2019, June 7). *Alexithymia**: Emotional blindness* http://www.alexithymia.us/test-alex.html

[bibr4-2041669520913319] Allgöwer K. Kern C. Hermsdörfer J. (2017). Predictive and reactive grip force responses to rapid load increases in people with multiple sclerosis. Archives of Physical Medicine and Rehabilitation, 98(3), 525–533. 27619952 10.1016/j.apmr.2016.08.465

[bibr6-2041669520913319] Bareket-Bojmel L. Moran S. Shahar G. (2016). Strategic self-presentation on Facebook: Personal motives and audience response to online behavior. Computers in Human Behavior, 55, 788–795.

[bibr8-2041669520913319] Baumeister R. F. Vohs K. D. Funder D. C. (2007). Psychology as the science of self-reports and finger movements: Whatever happened to actual behavior? Perspectives on Psychological Science, 2(4), 396–403. 26151975 10.1111/j.1745-6916.2007.00051.x

[bibr9-2041669520913319] Berry D. A. Stangl D. (2018). Bayesian biostatistics. CRC Press.

[bibr10-2041669520913319] Berryman C. McAuley J. H. Moseley L. G. (2012). Sphere 12 screening questionnaire. Journal of Physiotherapy, 58(4), 273. 23177234 10.1016/S1836-9553(12)70133-9

[bibr11-2041669520913319] Bilalpur M. Kia S. M. Chawla M. Chua T. S. Subramanian R. (2017, November). Gender and emotion recognition with implicit user signals. In *Proceedings of the 19th ACM International Conference on Multimodal Interaction* (pp. 379–387). ACM.

[bibr12-2041669520913319] Bradley M. M. Lang P. J. (2000). Measuring emotion: Behavior, feeling, and physiology. Cognitive Neuroscience of Emotion, 25, 49–59.

[bibr13-2041669520913319] Braunstein L. M. Gross J. J. Ochsner K. N. (2017). Explicit and implicit emotion regulation: A multi-level framework. Social Cognitive and Affective Neuroscience, 12(10), 1545–1557. 28981910 10.1093/scan/nsx096 PMC5647798

[bibr14-2041669520913319] Brooks S. J. Savov V. Allzén E. Benedict C. Fredriksson R. Schiöth H. B. (2012). Exposure to subliminal arousing stimuli induces robust activation in the amygdala, hippocampus, anterior cingulate, insular cortex and primary visual cortex: A systematic meta-analysis of fMRI studies. NeuroImage, 59(3), 2962–2973. 22001789 10.1016/j.neuroimage.2011.09.077

[bibr15-2041669520913319] Burr V. (2015). Social constructionism. Routledge.

[bibr16-2041669520913319] Campanella S. Chrysochoos A. Bruyer R. (2001). Categorical perception of facial gender information: Behavioural evidence and the face-space metaphor. Visual Cognition, 8(2), 237–262.

[bibr17-2041669520913319] Colonius H. Diederich A. (2017). Measuring multisensory integration: From reaction times to spike counts. Scientific Reports, 7(1), 3023. 28596602 10.1038/s41598-017-03219-5 PMC5465073

[bibr18-2041669520913319] Deacon, R. M. (2013). Measuring the strength of mice. *JoVE (Journal of Visualized Experiments)*, (76), e2610. 10.3791/2610 PMC3725666 23770643

[bibr19-2041669520913319] Dehaene S. Changeux J. P. Naccache L. Sackur J. Sergent C. (2006). Conscious, preconscious, and subliminal processing: A testable taxonomy. Trends in Cognitive Sciences, 10(5), 204–211. 16603406 10.1016/j.tics.2006.03.007

[bibr20-2041669520913319] Dienes Z. (2014). Using Bayes to get the most out of non-significant results. Frontiers in Psychology, 5, 781. 25120503 10.3389/fpsyg.2014.00781 PMC4114196

[bibr21-2041669520913319] Dienes Z. (2016). How Bayes factors change scientific practice. Journal of Mathematical Psychology, 72, 78–89.

[bibr22-2041669520913319] Dietrich J. Viljaranta J. Moeller J. Kracke B. (2017). Situational expectancies and task values: Associations with students’ effort. Learning and Instruction, 47, 53–64.

[bibr23-2041669520913319] Ellis N. C. (2002). Frequency effects in language processing: A review with implications for theories of implicit and explicit language acquisition. Studies in Second Language Acquisition, 24(2), 143–188.

[bibr24-2041669520913319] Evans A. M. Dillon K. D. Rand D. G. (2015). Fast but not intuitive, slow but not reflective: Decision conflict drives reaction times in social dilemmas. Journal of Experimental Psychology: General, 144(5), 951. 26413891 10.1037/xge0000107

[bibr25-2041669520913319] Faul F. Erdfelder E. Buchner A. Lang A. G. (2009). Statistical power analyses using G* Power 3.1: Tests for correlation and regression analyses. Behavior Research Methods, 41(4), 1149–1160. 19897823 10.3758/BRM.41.4.1149

[bibr26-2041669520913319] Fawcett T. (2006). An introduction to ROC analysis. Pattern Recognition Letters, 27(8), 861–874.

[bibr27-2041669520913319] Greenwald A. G. McGhee D. E. Schwartz J. L. (1998). Measuring individual differences in implicit cognition: The implicit association test. Journal of Personality and Social Psychology, 74(6), 1464. 9654756 10.1037//0022-3514.74.6.1464

[bibr28-2041669520913319] Gur R. C. Sara R. Hagendoorn M. Marom O. Hughett P. Macy L. Turner T. Bajcsy R. Posner A. Gur R. E. (2002). A method for obtaining 3-dimensional facial expressions and its standardization for use in neurocognitive studies. Journal of Neuroscience Methods, 115(2), 137–143. 11992665 10.1016/s0165-0270(02)00006-7

[bibr29-2041669520913319] Hellmann E. (2016). *Keeping up appearances: Perfectionism and perfectionistic self-presentation on social media*. scholarship.depauw.edu

[bibr30-2041669520913319] Hyde J. S. Bigler R. S. Joel D. Tate C. C. van Anders S. M. (2019). The future of sex and gender in psychology: Five challenges to the gender binary. American Psychologist, 74(2), 171. 30024214 10.1037/amp0000307

[bibr31-2041669520913319] Jarosz A. F. Wiley J. (2014). What are the odds? A practical guide to computing and reporting Bayes factors. The Journal of Problem Solving, 7(1), 2.

[bibr32-2041669520913319] Jurafsky D. (2000). Speech & language processing. Pearson Education India.

[bibr33-2041669520913319] Kaklauskas, A., Krutinis, M., & Seniut, M. (2009, July). Biometric mouse intelligent system for student’s emotional and examination process analysis. In *2009 Ninth IEEE International Conference on Advanced Learning Technologies* (pp. 189–193). IEEE

[bibr34-2041669520913319] Kim Y. Woo J. Woo M. (2017). Effects of stress and task difficulty on working memory and cortical networking. Perceptual and Motor Skills, 124, 1194–1210. 28942702 10.1177/0031512517732851

[bibr35-2041669520913319] Kooij, J. J. S., & Francken, M. H. (2010). *Diagnostic interview for ADHD in adults (DIVA)*. Hague: DIVA Foundation.

[bibr36-2041669520913319] Kopera M. Suszek H. Bonar E. Myszka M. Gmaj B. Ilgen M. Wojnar M. (2015). Evaluating explicit and implicit stigma of mental illness in mental health professionals and medical students. Community Mental Health Journal, 51(5), 628–634. 25535045 10.1007/s10597-014-9796-6 PMC4475542

[bibr37-2041669520913319] Kosakowska-Berezecka N. Jurek P. Besta T. Badowska S. (2017). Self-presentation strategies, fear of success and anticipation of future success among university and high school students. Frontiers in Psychology, 8, 1884. 29163271 10.3389/fpsyg.2017.01884 PMC5663907

[bibr38-2041669520913319] LeBel E. P. Paunonen S. V. (2011). Sexy but often unreliable: The impact of unreliability on the replicability of experimental findings with implicit measures. Personality and Social Psychology Bulletin, 37(4), 570–583. 21441219 10.1177/0146167211400619

[bibr39-2041669520913319] Lefly D. L. Pennington B. F. (2000). Reliability and validity of the adult reading history questionnaire. Journal of Learning Disabilities, 33(3), 286–296. 15505966 10.1177/002221940003300306

[bibr40-2041669520913319] Lewinski P. (2015). Automated facial coding software outperforms people in recognizing neutral faces as neutral from standardized datasets. Frontiers in Psychology, 6, 1386. 26441761 10.3389/fpsyg.2015.01386 PMC4565996

[bibr41-2041669520913319] Motta‐Mena N. V. Scherf K. S. (2017). Pubertal development shapes perception of complex facial expressions. Developmental Science, 20(4), e12451. 10.1111/desc.12451 27321445

[bibr42-2041669520913319] Noldus. (2018, July 23). *Reference manual* http://www.noldus.com/human-behaviorresearch/products/facereader

[bibr46-2041669520913319] Perkins A. M. Ettinger U. Davis R. Foster R. Williams S. C. Corr P. J. (2009). Effects of lorazepam and citalopram on human defensive reactions: Ethopharmacological differentiation of fear and anxiety. Journal of Neuroscience, 29(40), 12617–12624. 19812336 10.1523/JNEUROSCI.2696-09.2009 PMC6665108

[bibr48-2041669520913319] Pessoa L. Adolphs R. (2010). Emotion processing and the amygdala: From a ‘low road’ to ‘many roads’ of evaluating biological significance. Nature Reviews Neuroscience, 11(11), 773. 20959860 10.1038/nrn2920 PMC3025529

[bibr50-2041669520913319] Ryoo M. Burlingham C. Roth Z. Mirbagheri S. Heeger D. J. Merriam E. (2018). A widespread task-related hemodynamic response in human V1 is modulated by task difficulty. Journal of Vision, 18(10), 1261–1261.

[bibr52-2041669520913319] Schnabel K. Asendorpf J. B. Greenwald A. G. (2008). Assessment of individual differences in implicit cognition: A review of IAT measures. European Journal of Psychological Assessment, 24(4), 210–217.

[bibr53-2041669520913319] Schunk D. H. Greene J. A. (2017). Handbook of self-regulation of learning and performance. Routledge.

[bibr54-2041669520913319] Stanley D. Phelps E. Banaji M. (2008). The neural basis of implicit attitudes. Current Directions in Psychological Science, 17(2), 164–170.

[bibr55-2041669520913319] Strack F. Deutsch R. (2004). Reflective and impulsive determinants of social behavior. Personality and Social Psychology Review, 8(3), 220–247. 15454347 10.1207/s15327957pspr0803_1

[bibr56-2041669520913319] Suslow T. Ihme K. Quirin M. Lichev V. Rosenberg N. Bauer J. Lobsien D. (2015). Implicit affectivity and rapid processing of affective body language: An fMRI study. Scandinavian Journal of Psychology, 56(5), 545–552. 26032148 10.1111/sjop.12227

[bibr57-2041669520913319] Tsikandilakis, M., Bali, P., & Chapman, P. (2019a). Beauty is in the eye of the beholder: The appraisal of facial attractiveness and its relation to conscious awareness. *Perception, 48*(1), 72–92. 10.1177/0301006618813035 30567468

[bibr58-2041669520913319] Tsikandilakis M. Bali P. Derrfuss J. Chapman P. (2019b). The unconscious mind: From classical theoretical controversy to controversial contemporary research and a practical illustration of the “error of our ways”. Consciousness and Cognition, 74, 102771. 31299420 10.1016/j.concog.2019.102771

[bibr59-2041669520913319] Tsikandilakis, M., Bali, P., Derrfuss, J., & Chapman, P. (2019). “I can see you; I can feel it; and vice-versa”: consciousness and its relation to emotional physiology. *Cognition and Emotion*, 1–13. 10.1080/02699931.2019.1646710 31354042

[bibr60-2041669520913319] Tsikandilakis, M., Bali, P., Derrfuss, J., & Chapman, P. (2019). Anger and hostility: are they different? An analytical exploration of facial-expressive differences, and physiological and facial-emotional responses. *Cognition and Emotion*, 1–15. 10.1080/02699931.2019.1664415 31522602

[bibr61-2041669520913319] Tsikandilakis M. Chapman P. (2018). Skin-conductance responses to masked emotional faces are modulated by hit rate but not signal detection theory adjustments for subjective differences in the detection threshold. Perception, 47(4), 432–450. 29466909 10.1177/0301006618760738

[bibr62-2041669520913319] Tsikandilakis M. Chapman P. Peirce J. (2018). Target meta-awareness is a necessary condition for physiological responses to masked emotional faces: Evidence from combined skin conductance and heart rate assessment. Consciousness and Cognition, 58, 75–89. 29239755 10.1016/j.concog.2017.10.013

[bibr63-2041669520913319] Tsikandilakis, M., Kausel, L., Boncompte, G., Yu, Z., Oxner, M., Lanfranco, R., … & Tong, E. M. W. (2019). “There Is No Face Like Home”: Ratings for Cultural Familiarity to Own and Other Facial Dialects of Emotion With and Without Conscious Awareness in a British Sample. *Perception, 48*(10), 918–947. 10.1177/0301006619867865 31451029

[bibr64-2041669520913319] van der Ploeg M. M. Brosschot J. F. Versluis A. Verkuil B. (2017). Peripheral physiological responses to subliminally presented negative affective stimuli: A systematic review. Biological Psychology, 129, 131–153. 28843622 10.1016/j.biopsycho.2017.08.051

[bibr65-2041669520913319] Yablonski M. Polat U. Bonneh Y. S. Ben-Shachar M. (2017). Microsaccades are sensitive to word structure: A novel approach to study language processing. Scientific Reports, 7(1), 3999. 28638094 10.1038/s41598-017-04391-4 PMC5479819

[bibr66-2041669520913319] Zhang J. Mueller S. T. (2005). A note on ROC analysis and non-parametric estimate of sensitivity. Psychometrika, 70(1), 203–212.

