# Supplementary material for: “The Harder One Tries …”: Findings and Insights From the Application of Covert Response Pressure Assessment Technology in Three Studies of Visual Perception
Source: Iperception. 2020 Apr 20;11(2):2041669520913319. doi: 10.1177/2041669520913319 (PMC7171999; doi:10.1177/2041669520913319)

Supplementary Material

1.1: Normality Tests Stage One

Force Pressure Normality Tests per Interval

		10% Male	30% Male	50% Male	70% Male	90% Male
N		54	54	54	54	54
Normal Parameters[a, b]	Mean	.1138	.0620	.0499	.0631	.1099
	Std. Deviation	.04226	.02556	.02594	.02723	.03884
Most Extreme Differences	Absolute	.114	.099	.101	.118	.103
	Positive	.114	.099	.094	.118	.085
	Negative	-.106	-.068	-.101	-.071	-.103
Kolmogorov-Smirnov Z		.839	.724	.742	.864	.753
Asymp. Sig. (2-tailed)		.482	.670	.640	.444	.622

a. Test distribution is Normal.

Release Times Normality Tests per Interval

		10% Male	30% Male	50% Male	70% Male	90% Male
N		54	54	54	54	54
Normal Parameters[a, b]	Mean	.1815	.2719	.3746	.2809	.1828
	Std. Deviation	.06822	.09002	.08364	.08319	.07546
Most Extreme Differences	Absolute	.076	.087	.088	.086	.097
	Positive	.067	.085	.062	.057	.067
	Negative	-.076	-.087	-.088	-.086	-.097
Kolmogorov-Smirnov Z		.555	.640	.645	.631	.711
Asymp. Sig. (2-tailed)		.917	.808	.800	.820	.692

a. Test distribution is Normal.

1.2: Normality Tests Stage Two

Force Pressure Normality Tests per Emotion

		Force Pressure Fearfull Faces	Force Pressure Sad Faces	Force Pressure Neutral Faces
N		48	48	48
Normal Parameters[a,b]	Mean	.2686250	.1236458	.1190625
	Std. Deviation	.08576902	.03679182	.03447071
Most Extreme Differences	Absolute	.115	.130	.101
	Positive	.115	.081	.075
	Negative	-.104	-.130	-.101
Kolmogorov-Smirnov Z		.798	.898	.701
Asymp. Sig. (2-tailed)		.547	.395	.710

a. Test distribution is Normal.

Release Times Normality Tests per Emotion

		Release Times Fearfull Faces	Release Times Sad Faces	Release Times Neutral Faces
N		48	48	48
Normal Parameters[a,b]	Mean	.2573542	.2507500	.2445625
	Std. Deviation	.04060892	.04145672	.03911408
Most Extreme Differences	Absolute	.140	.121	.093
	Positive	.093	.085	.086
	Negative	-.140	-.121	-.093
Kolmogorov-Smirnov Z		.973	.841	.647
Asymp. Sig. (2-tailed)		.300	.479	.797

a. Test distribution is Normal.

1.3: Study Three Normality

Force Pressure Normality Tests per Letter String

		Two Letters Force Pressure	Three Letters Force Pressure	Four Letter Force Pressure
N		60	60	60
Normal Parameters[a,b]	Mean	.1308	.1127	.1034
	Std. Deviation	.03548	.02922	.02771
Most Extreme Differences	Absolute	.132	.136	.124
	Positive	.087	.136	.087
	Negative	-.132	-.133	-.124
Kolmogorov-Smirnov Z		1.021	1.056	.957
Asymp. Sig. (2-tailed)		.248	.215	.319

a. Test distribution is Normal.

Release Times Normality Tests per Letter String

		Two Letters Release Times	Three Letters Release Times	Four Letter Release Times
N		60	60	60
Normal Parameters[a,b]	Mean	.3813	.5020	.6953
	Std. Deviation	.08985	.12433	.18847
Most Extreme Differences	Absolute	.068	.076	.129
	Positive	.062	.065	.077
	Negative	-.068	-.076	-.129
Kolmogorov-Smirnov Z		.524	.586	1.001
Asymp. Sig. (2-tailed)		.946	.882	.269

a. Test distribution is Normal.

2.1. Left-Handed Participants

Left-handed participants were not included in the current study. Pilot-testing handedness assessment including left-handed participants (n = 20) indicated that 65% (n = 13) of left-handed participants used their right hand and left hand interchangeably in different activities, such as using a mouse, writing, typing, texting and domestic activities, or were instructed at some stage of their educational upbringing to write with their non-dominant hand. To attain consistency in respect to hand-dominance in the current studies, we included right-handed participants only. Future studies could benefit from a dedicated exploration of left-handedness using the current assessment.

3.1 Stimuli Selection

See Fig. 4.

	**Session**					
	A (type): Mean (S.D.)		B (experience) Mean (S.D.)			
**Accuracy (%)**	Group AB	Group BA	Group AB	Group BA	Overall Session A	Overall Session B
Angry*	71.97 (9.97)	80.55 (5.27)	46.35 (11.25)	44.44 (8.07)	76.47 (8.79)	45 .29 (9.43)
Fearful	75 (8.01)	77.22 (6.66)	53.12 (7.98)	57.22 (11.48)	76.17 (7.18)	55.29 (9.91)
Disgusted	73.12 (10.66)	67.77 (8.7)	45 (8.45)	40.55 (7.26)	70.29 (9.75)	42.64 (7.92)
Happy	81.25 (9.54)	81.11 (10.24)	60 (11.95)	66.66 (12.74)	81.17 (9.6)	63.52 (12.47)
Sad	85.62 (5.62)	80 (9.35)	54.37 (8.63)	57.55 (7.94)	82.64 (8.12)	56.17 (8.2)
Neutral	88.75 (4.43)	85.55 (8.45)	66.9 (4.58)	70 (16.95)	87.05 (6.85)	68.52 (12.47)
**Intensity (1 - 10)**						
Angry	6.3 (.89)	6.02 (1.32)	7.35 (1.09)	7.13 (1.69)	6.15 (1.11)	7.23 (1.4)
Fearful	5.91 (.46)	5.48 (,63)	7.06 (.35)	6.71 (1.14)	5.68 (.58)	6.88 (.86)
Disgusted	5.46 (.34)	5.88 (.67)	6.43 (.26)	6.92 (1.01)	5.68 (.56)	6.69 (.77)
Happy	5.96 (.59)	5.87 (.45)	7.51 (1.01)	7.42 (.64)	5.91 (.5)	7.46 (.81)
Sad	4.88 (,48)	4.81 (.59)	5.06 (1.15)	5.26 (.77)	4.84 (.52)	5.16 (.94)
**Ambiguity (1 - 10)****						
Angry	5.62 (1.02)	5.66 (1.34)	4.63 (1.29)	3.5 (1.07)	5.64 (1.16)	4.03 (1.28)
Fearful	5.24 (1.13)	5.16 (.81)	4.19 (.73)	4.05 (1)	5.19 (.94)	4.12 (.86)
Disgusted	6.23 (.58)	5.29 (.51)	4.98 (.85)	4.49 (.32)	5.74 (.71)	4.72 (.65)
Happy	5.28 (.48)	5.2 (.56)	3.69 (.66)	3.61 (.55)	5.24 (.51)	3.64 (.59)
Sad	5.61 (.58)	5.52 (.48)	3.79 (.75)	3.96 (.65)	5.56 (.51)	3.88 (.68)
Neutral	4.38 (.6)	4.93 (.84)	2.93 (.96)	3.26 (.96)	4.67 (.77)	3.1 (.94)
**Accuracy Analysis (p values)**	(AT Task)					
(AE Task)	Angry	Fearful	Disgusted	Happy	Sad	Neutral
Angry*	.00***	.138	.365	.016	.005	.00
Fearful	.	.00***	.003	.65	1	.007
Disgusted		.	.00***	.003	.00	.00
Happy		.	.	.00***	1	1
Sad	.				.00***	.001
Neutral		.	.		.	.00***

4.1 Assessment of Awareness for Physiological Recording

To achieve participant unawareness concerning the physiological assessment the participants did not receive any instructions during the pre-experimental briefing, they did not receive any movement restriction feedback at any point before or during the experiments and they were informed before the experiment that there were no time restrictions for replying to the engagement tasks. Baseline force-pressure was measured during the training stages. In this stage participants were informed that an initial phase of the current study was to confirm that the responses are properly recorded and that they are aware of how to perform the experimental tasks. After the end of the experiment, participants were asked using a pre-debrief-form questionnaire whether they were aware of any recording of their responses. For Studies one and three no participants responded that they were aware of the recording of their responses. For study two, three participants (two males) replied “yes”. When asked further in a subsequent section of the pre-debriefing document, one (male) replied “the researcher could have been monitoring my responses” and two replied “camera recording during the experiment” and “my responses were monitored by a camera”. Both participants who replied that their expressions might have been subject to camera recording were further inquired concerning the reasons for their reply. They responded that they were previously part of an overt-explicit camera analysis emotional assessment study. All participants were informed after the experiment concerning the recording of their responses. All participants were kindly informed that it would to the benefit of future replications of the current design to not disclose the implicit assessment during the current experiment. All participants were explicit informed that they were not under any kind of obligation to follow this suggestion.

5.1 Native and non-Native Speakers

		Sum of Squares	Mean Square	F	Sig.
Three_F	Between Groups	.000	.000	.062	.940
	Within Groups	.045	.001		
	Total	.045			
Three_RT	Between Groups	.001	.000	.036	.965
	Within Groups	.773	.014		
	Total	.774			
Three_CF	Between Groups	.406	.203	.211	.811
	Within Groups	54.927	.964		
	Total	55.333			
Two_F	Between Groups	.001	.000	.384	.683
	Within Groups	.073	.001		
	Total	.074			
Three_FP	Between Groups	.000	.000	.004	.996
	Within Groups	.050	.001		
	Total	.050			
Four_FP	Between Groups	.006	.003	4.221	.020
	Within Groups	.042	.001		
	Total	.048			
Two_RT	Between Groups	.002	.001	.098	.907
	Within Groups	.475	.008		
	Total	.476			
Three_CRT	Between Groups	.001	.000	.017	.983
	Within Groups	.911	.016		
	Total	.912			
Four_RT	Between Groups	.006	.003	.079	.924
	Within Groups	2.090	.037		
	Total	2.096			
Two_CF	Between Groups	.846	.423	.578	.565
	Within Groups	41.723	.732		
	Total	42.569			
Three_CNF	Between Groups	.451	.226	.179	.836
	Within Groups	71.833	1.260		
	Total	72.285			
Four_CNF	Between Groups	.047	.024	.018	.982
	Within Groups	76.249	1.338		
	Total	76.296			
RTTW	Between Groups	.198	.099	1.996	.145
	Within Groups	2.821	.049		
	Total	3.018			

	Between Groups	.030	.015	.294	.747
RTTH	Within Groups	2.954	.052		
	Total	2.984			
	Between Groups	.048	.024	.396	.675
RTFR	Within Groups	3.417	.060		
	Total	3.465			

6.1: Summary of Pilot Task

Summary Responses to Backwards Masked Emotional faces when participants were briefed concerning the recording of their responses.

Mean Z -Scores for Engagement Task Force Pressure

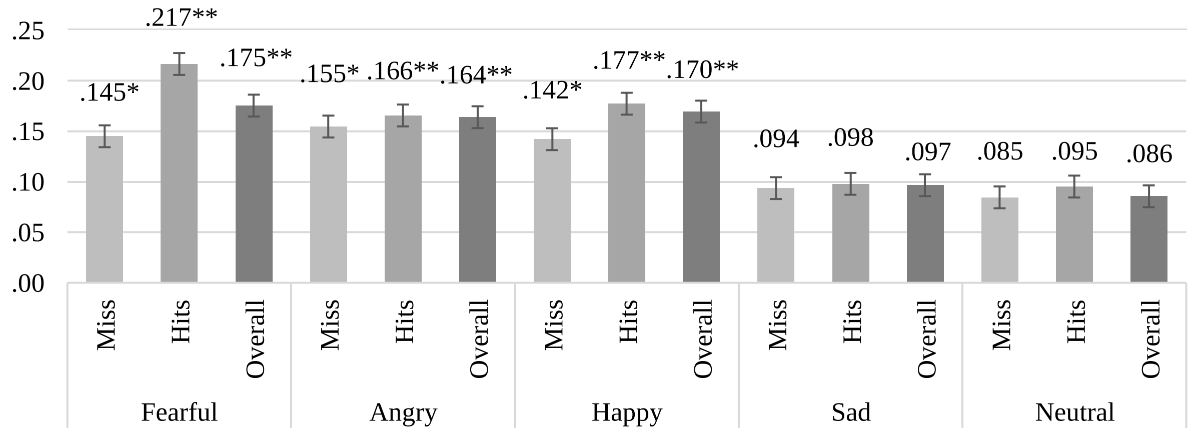

Note. Engagement task force pressure in standardised Z-scores units of pressure (micro-Newtons) based on the preliminary training task. Bars show standard error of the mean. * indicates p < .01, ** indicates p < .001.

Summary Responses and Correlation with Skin Conductance during Emotional Morphing

Neutral	Fear	Force-Pressure (Z-scores)	Correlation r (SCR)	Click-release-time (Seconds)
100%	0%	.04	.89	.12
80%	20%	.017	.91	.19
60%	40%	.069	.91	.53
40%	60%	.103	.92	.61
20%	80%	.198	.96	.34
0%	100%	.245	.92	.24

7.1 Developed time-protocol, parallel input, coding scheme, processing and formula material

See https://osf.io/u5eac/

8.1: Subliminality

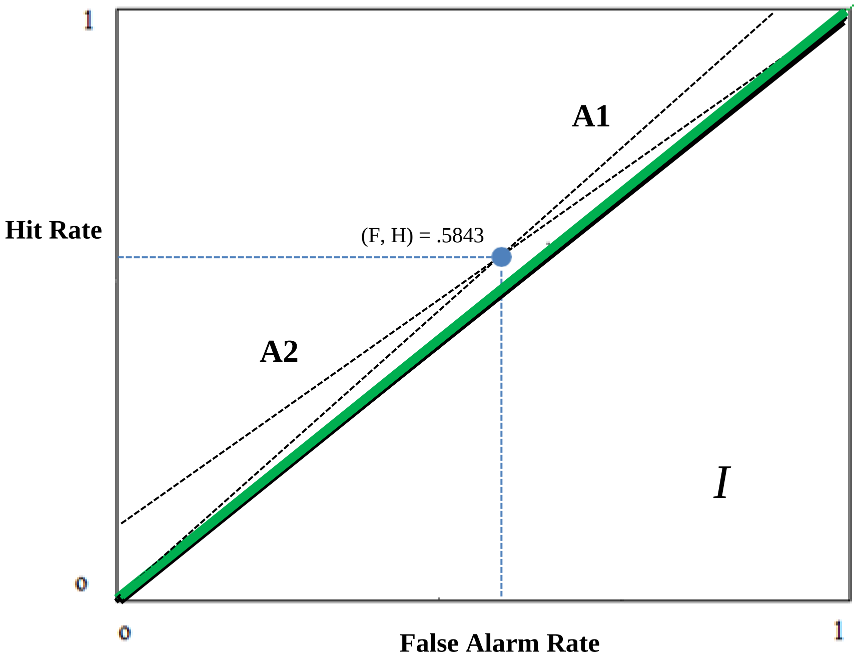

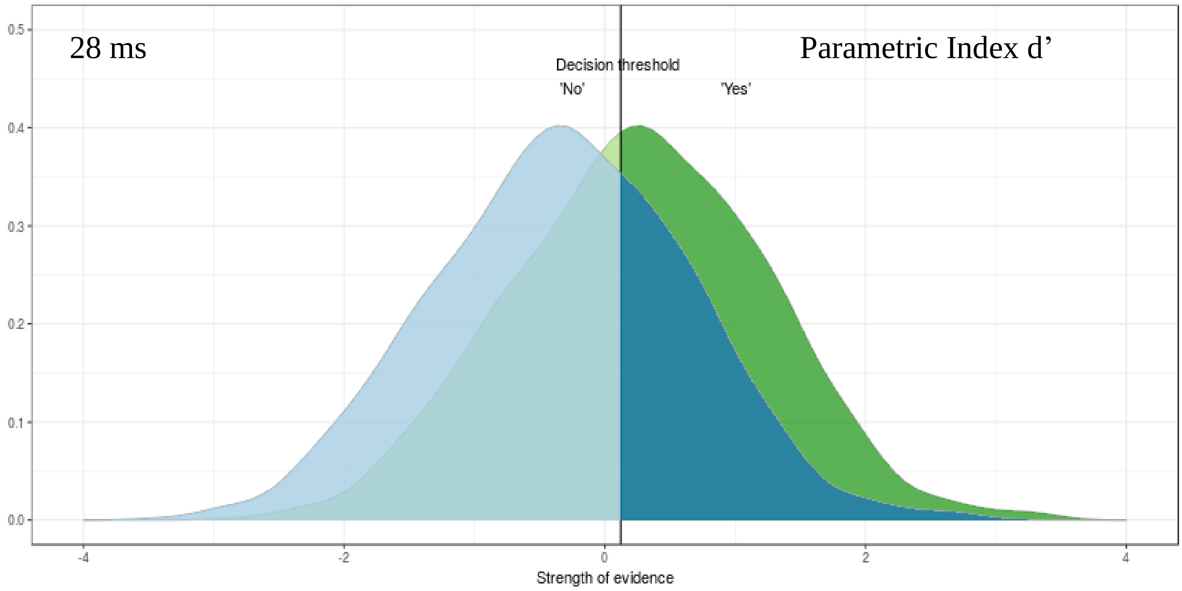

ROC One: Detection Performance for 27.78 ms. Programmed according to a single threshold design including A1 and A2 sectors for possible range of varying (F,H) characteristics for detection performance. Also provided using parametric index d'.

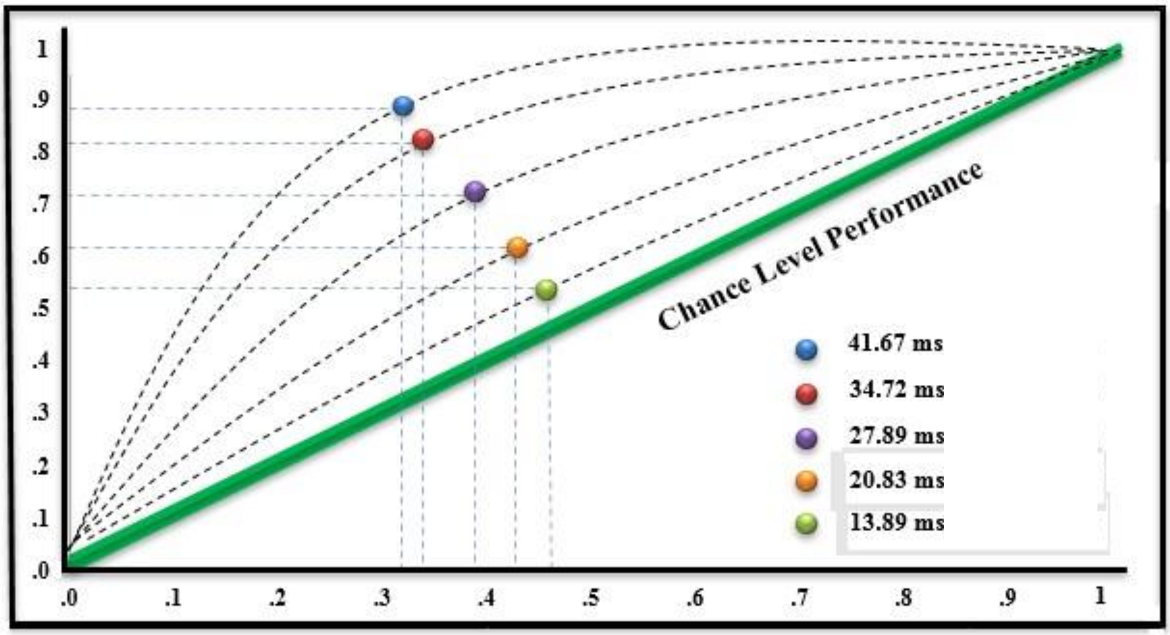
Chance Level Performance
41.67 ms
34.72 ms
27.89 ms
20.83 ms
13.89 ms
1
.9
.8
.7
.6
.5
.4
.3
.2
.1
.0
.0
.1
.2
.3
.4
.5
.6
.7
.8
.9
1

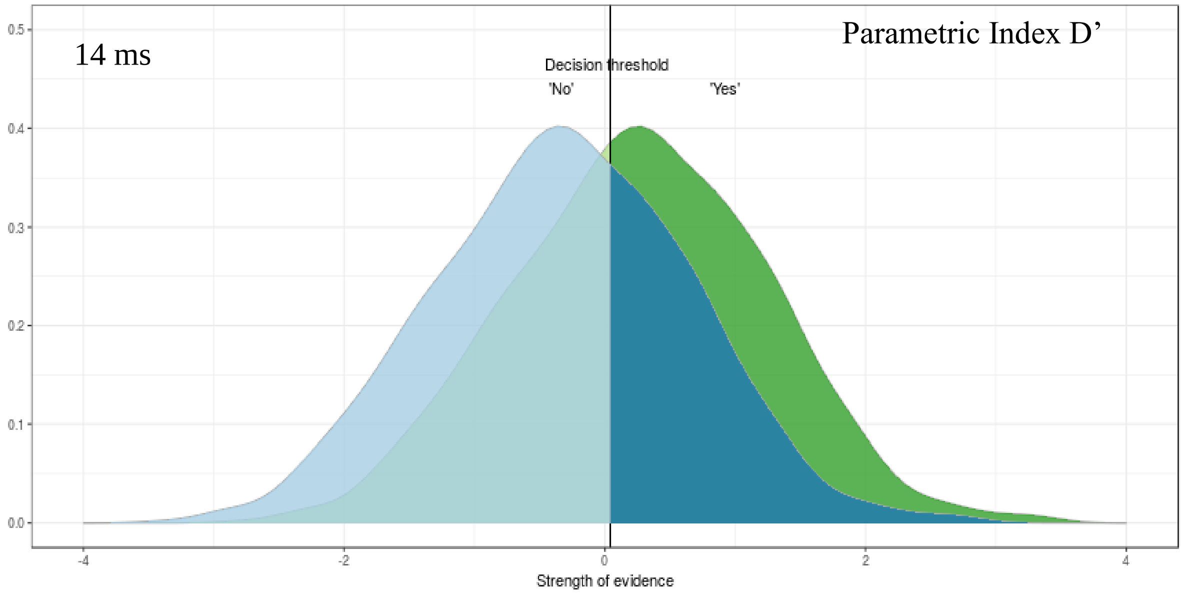
14 ms
Parametric Index D'
Decision threshold
'No'
'Yes'
0.5
0.4
0.3
0.2
0.1
0.0
-4
-2
0
2
4
Strength of evidence

Confidence-Subliminality

Measure: MEASURE_1

Source		Type III Sum of Squares	df	Mean Square	F	Sig.	Partial Eta Squared
Em	Sphericity Assumed	.609	2	.305	.181	.835	.004
	Greenhouse-Geisser	.609	1.991	.306	.181	.834	.004
	Huynh-Feldt	.609	2.000	.305	.181	.835	.004
	Lower-bound	.609	1.000	.609	.181	.672	.004
Error(Em)	Sphericity Assumed	154.845	92	1.683			
	Greenhouse-Geisser	154.845	91.599	1.690			
	Huynh-Feldt	154.845	92.000	1.683			
	Lower-bound	154.845	46.000	3.366			
Det	Sphericity Assumed	.048	1	.048	.032	.859	.001
	Greenhouse-Geisser	.048	1.000	.048	.032	.859	.001
	Huynh-Feldt	.048	1.000	.048	.032	.859	.001
	Lower-bound	.048	1.000	.048	.032	.859	.001
Error(Det)	Sphericity Assumed	68.500	46	1.489			
	Greenhouse-Geisser	68.500	46.000	1.489			
	Huynh-Feldt	68.500	46.000	1.489			
	Lower-bound	68.500	46.000	1.489			
Em * Det	Sphericity Assumed	.639	2	.320	.146	.864	.003
	Greenhouse-Geisser	.639	1.957	.327	.146	.860	.003
	Huynh-Feldt	.639	2.000	.320	.146	.864	.003
	Lower-bound	.639	1.000	.639	.146	.704	.003
Error(Em*Det)	Sphericity Assumed	201.428	92	2.189			
	Greenhouse-Geisser	201.428	90.016	2.238			
	Huynh-Feldt	201.428	92.000	2.189			
	Lower-bound	201.428	46.000	4.379			

Release-Time Subliminality

Measure: MEASURE_1

Source		Type III Sum of Squares	df	Mean Square	F	Sig.	Partial Eta Squared
Em	Sphericity Assumed	.001	2	.001	.051	.951	.001
	Greenhouse-Geisser	.001	1.935	.001	.051	.947	.001
	Huynh-Feldt	.001	2.000	.001	.051	.951	.001
	Lower-bound	.001	1.000	.001	.051	.823	.001
Error(Em)	Sphericity Assumed	.977	92	.011			
	Greenhouse-Geisser	.977	89.031	.011			
	Huynh-Feldt	.977	92.000	.011			
	Lower-bound	.977	46.000	.021			
Det	Sphericity Assumed	.000	1	.000	.009	.926	.000
	Greenhouse-Geisser	.000	1.000	.000	.009	.926	.000
	Huynh-Feldt	.000	1.000	.000	.009	.926	.000
	Lower-bound	.000	1.000	.000	.009	.926	.000
Error(Det)	Sphericity Assumed	.669	46	.015			
	Greenhouse-Geisser	.669	46.000	.015			
	Huynh-Feldt	.669	46.000	.015			
	Lower-bound	.669	46.000	.015			
Em * Det	Sphericity Assumed	.009	2	.005	.441	.645	.009
	Greenhouse-Geisser	.009	1.985	.005	.441	.643	.009
	Huynh-Feldt	.009	2.000	.005	.441	.645	.009
	Lower-bound	.009	1.000	.009	.441	.510	.009
Error(Em*Det)	Sphericity Assumed	.978	92	.011			
	Greenhouse-Geisser	.978	91.310	.011			
	Huynh-Feldt	.978	92.000	.011			
	Lower-bound	.978	46.000	.021			

9.1 Python Click-Monitoring Basic Function

```
import win32api
import time

def LeftClickPressTime():
   state_left = win32api.GetKeyState(0x01) # Left button down = 0 or 1. Button up = -127 or
-128
   while True:
      a = win32api.GetKeyState(0x01)
      if a != state_left: # Button state changed
         state_left = a
         if a < 0:
            start_time = time.time()
         else:
            global end_time
            end_time = time.time() - start_time
            return end_time
```

9.2: Python Keyboard Monitoring Basic Function

```
from pynput import keyboard
import time
def callb(key): #what to do on key-release
   ti1 = str(time.time() - t)[0:5] #converting float to str, slicing the float
   print("The key",key," is pressed for",ti1,'seconds')
   return False #stop detecting more key-releases
def callb1(key): #what to do on key-press
   return False #stop detecting more key-presses
try:
   while True:
      with keyboard.Listener(on_press=callb1) as listener1: # setting code for listening key-
press
         listener1.join()
      t = time.time() # reading time in sec
      with keyboard.Listener(on_release=callb) as listener: # setting code for listening key-
release
         listener.join()
except KeyboardInterrupt:
   pass
```

10.1 Release Time

Source		Type III Sum of Squares	df	Mean Square	F	Sig.	Partial Eta Squared
factor1	Sphericity Assumed	1.394	4	.349	1681.583	.000	.969
	Greenhouse-Geisser	1.394	2.487	.561	1681.583	.000	.969
	Huynh-Feldt	1.394	2.620	.532	1681.583	.000	.969
	Lower-bound	1.394	1.000	1.394	1681.583	.000	.969
Error(factor1)	Sphericity Assumed	.044	212	.000			
	Greenhouse-Geisser	.044	131.836	.000			
	Huynh-Feldt	.044	138.840	.000			
	Lower-bound	.044	53.000	.001			

11.1 Split-Half Correlation Analysis for Stage One: Reliability for Release Times and Force Pressure

Reliability Statistics

Cronbach's Alpha	Part 1	Value	.982
		N of Items	5[a]
	Part 2	Value	.993
		N of Items	5[b]
	Total N of Items		10
Correlation Between Forms			-.998
Spearman-Brown Coefficient	Equal Length		-816.386[c]
	Unequal Length		-.999[c]
Guttman Split-Half Coefficient			-4.356

a. The items are: Force Pressure for Ten Percent Male, Thirty Percent Male, Fifty Percent Male. Seventy Percent Male, Ninety Percent Male

b. The items are: Release Times for Ten Percent Male, Thirty Percent Male, Fifty Percent Male. Seventy Percent Male, Ninety Percent Male

c. The correlation between forms (halves) of the test is negative.

11.2 Split-Half Correlation Analysis for Stage Two: Reliability for Release Times and Force Pressure

Reliability Statistics

Cronbach's Alpha	Part 1	Value	.678
		N of Items	3[a]
	Part 2	Value	.662
		N of Items	3[b]
	Total N of Items		6
Correlation Between Forms			-.604
Spearman-Brown Coefficient	Equal Length		-3.050[c]
	Unequal Length		-.753[c]
Guttman Split-Half Coefficient			-2.614

a. The items are: Force Pressure for Fearful, Sad and Neutral Faces.

b. The items are: Release Times for Fearful, Sad and Neutral Faces.

c. The correlation between forms (halves) of the test is negative.

11.3 Split-Half Correlation Analysis for Stage Three: Reliability for Release Times and Force Pressure

Reliability Statistics

Cronbach's Alpha	Part 1	Value	.987
		N of Items	3[a]
	Part 2	Value	.948
		N of Items	3[b]
	Total N of Items		6
Correlation Between Forms			-.994
Spearman-Brown Coefficient	Equal Length		-357.645[c]
	Unequal Length		-.997[c]
Guttman Split-Half Coefficient			-1.553

a. The items are: Force Pressure for Two, Three and Four Letter Strings.

b. The items are: Release Times for Two, Three and Four Letter Strings

c. The correlation between forms (halves) of the test is negative.

12.1: Force Pressure and Gender Characteristics

Force Pressure Responses per Percentage of Male Characteristics

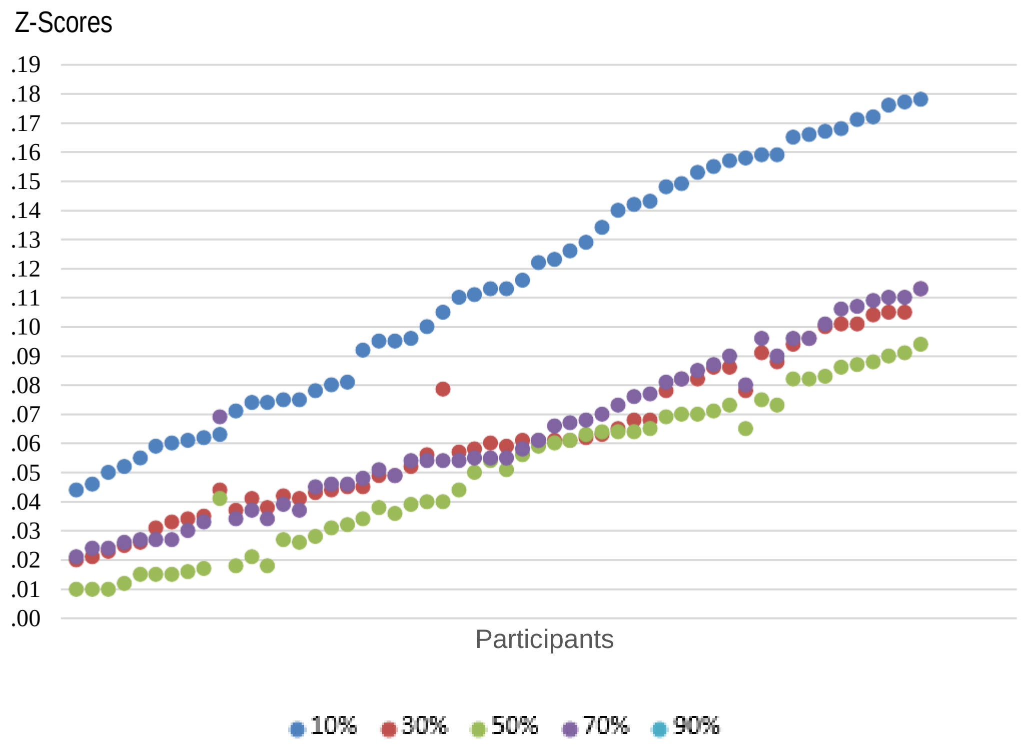

Release Responses per Percentage of Male Characteristics

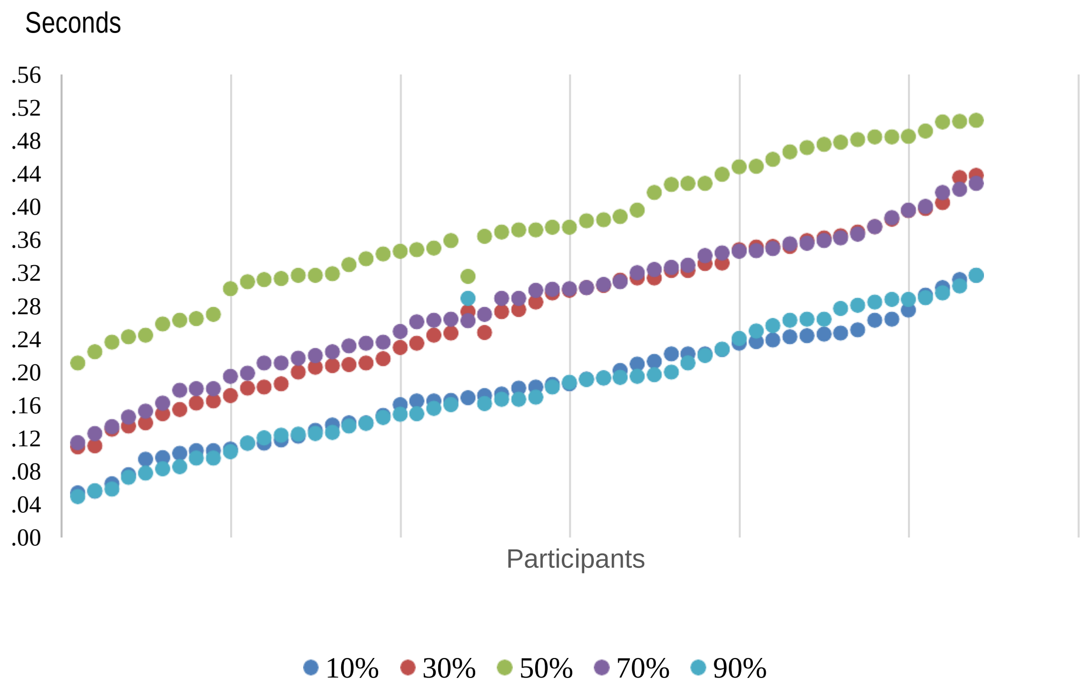

12.2: Force Pressure and Emotion

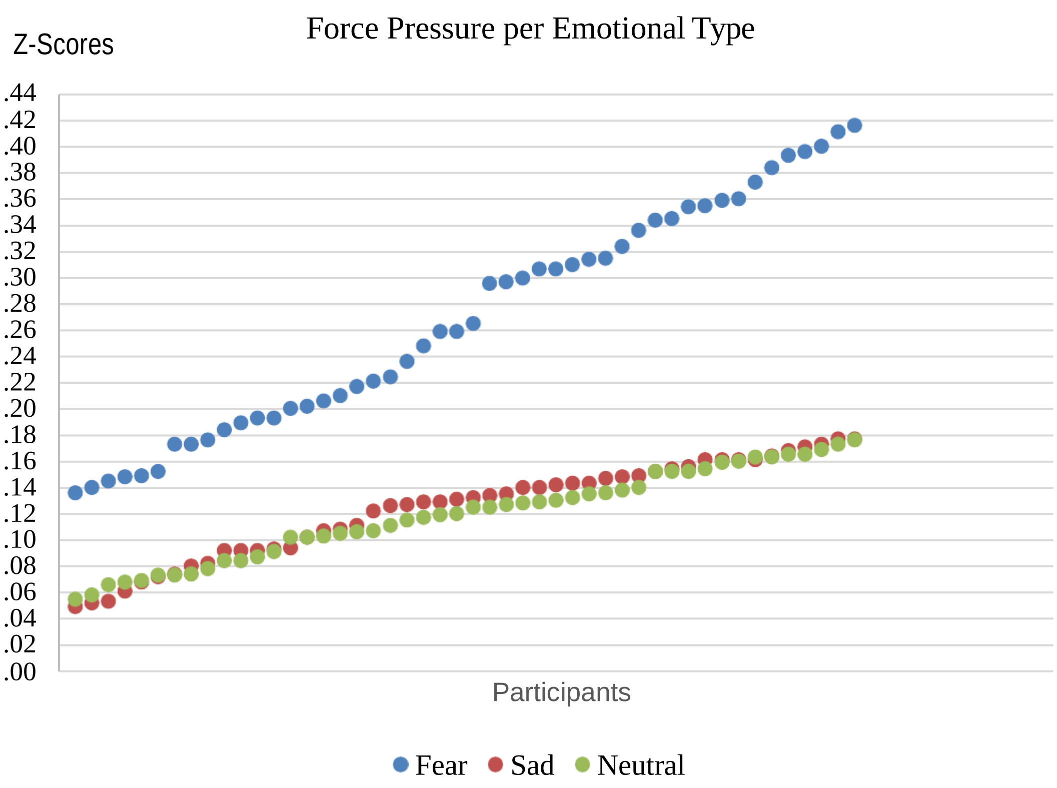

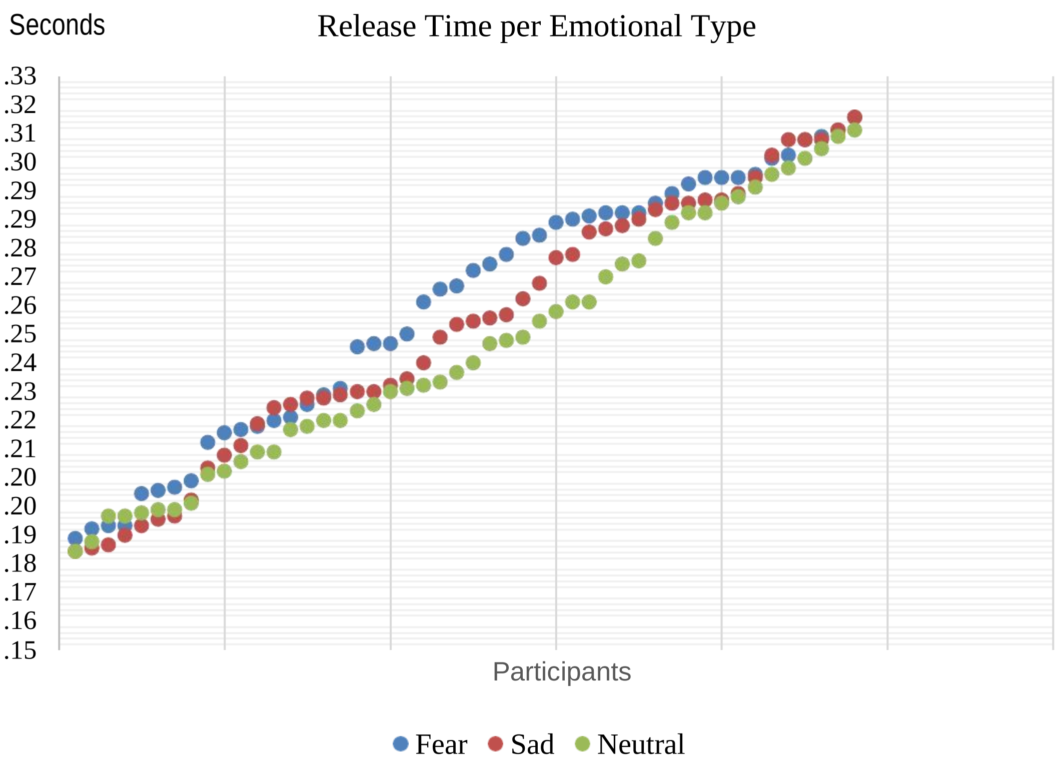

12.3 Force Pressure and Letter Length

12.3: Force Pressure and Letter Length

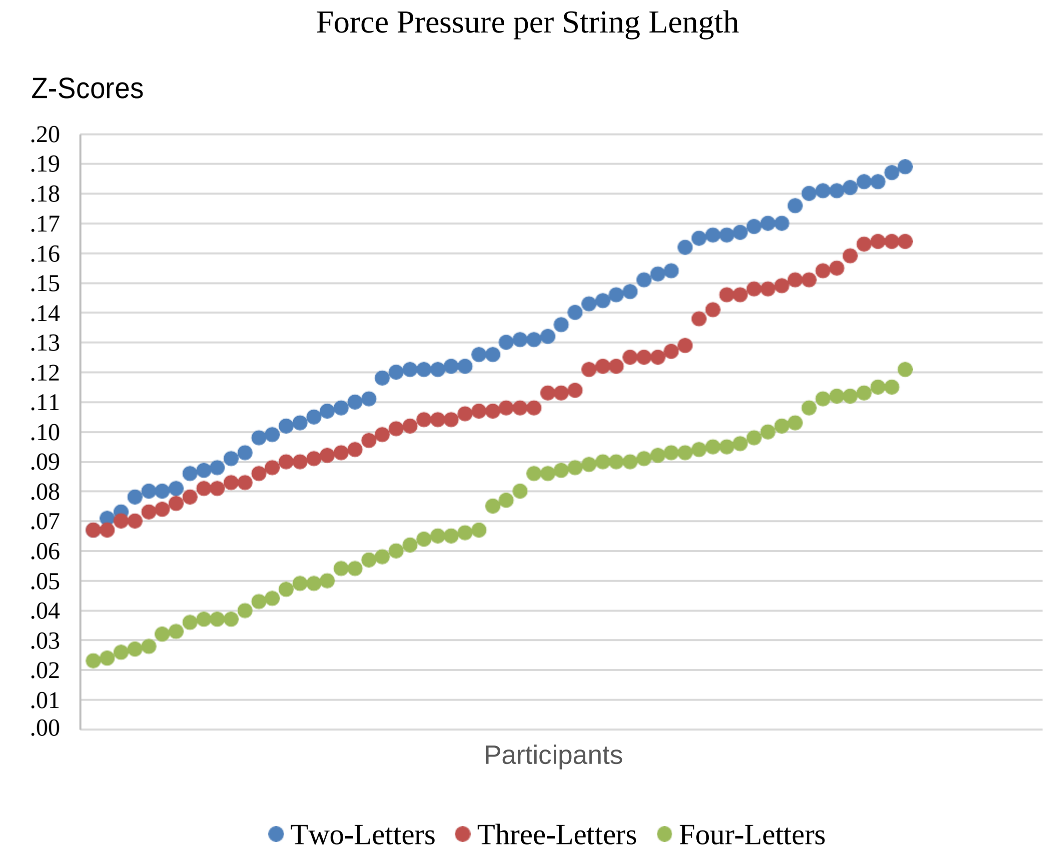

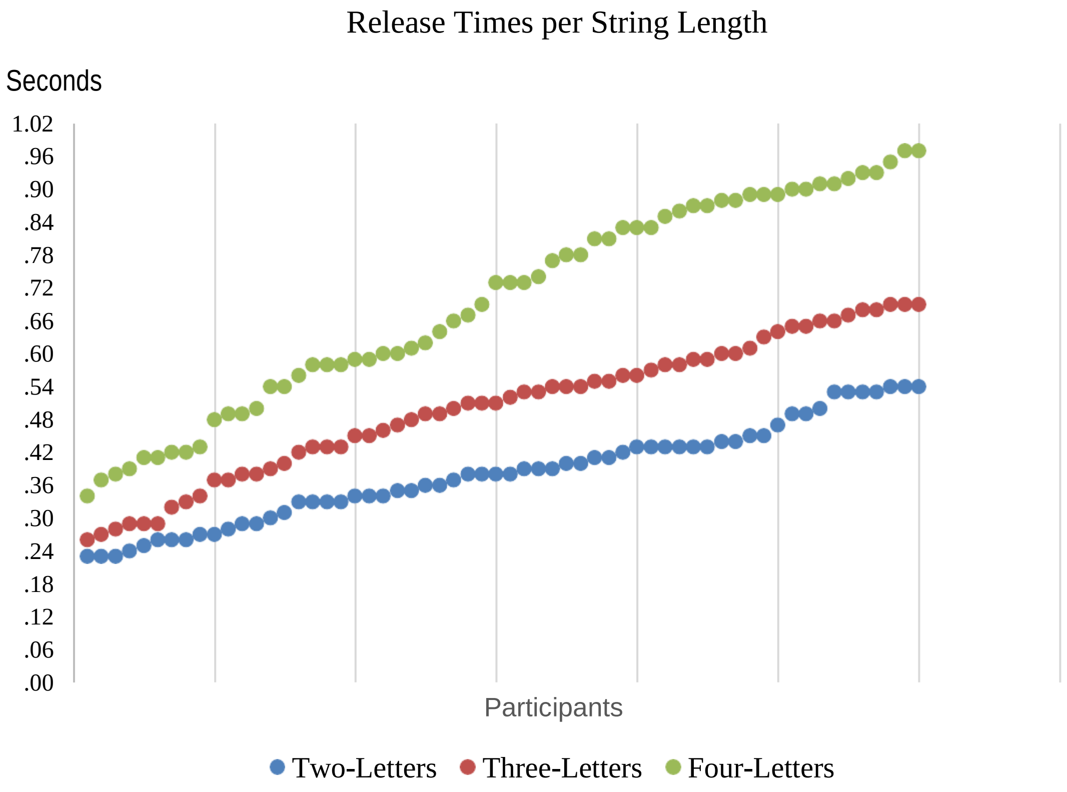

Supplement: sj-pdf-1-ipe-10.1177_2041669520913319 - Supplemental material for “The Harder One Tries …”: Findings and Insights From the Application of Covert Response Pressure Assessment Technology in Three Studies of Visual Perception [file sj-pdf-1-ipe-10.1177_2041669520913319.pdf]
